# Liver nucleotide biosynthesis is linked to protection from vascular complications in individuals with long-term type 1 diabetes

**DOI:** 10.1038/s41598-020-68130-y

**Published:** 2020-07-14

**Authors:** Ruchi Jain, Türküler Özgümüş, Troels Mygind Jensen, Elsa du Plessis, Magdalena Keindl, Cathrine Laustrup Møller, Henrik Falhammar, Thomas Nyström, Sergiu-Bogdan Catrina, Gun Jörneskog, Leon Eyrich Jessen, Carol Forsblom, Jani K. Haukka, Per-Henrik Groop, Peter Rossing, Leif Groop, Mats Eliasson, Björn Eliasson, Kerstin Brismar, Mahmoud Al-Majdoub, Peter M. Nilsson, Marja-Riitta Taskinen, Ele Ferrannini, Peter Spégel, Tore Julsrud Berg, Valeriya Lyssenko

**Affiliations:** 1 Department of Clinical Science/Diabetes and Endocrinology, Lund University Diabetes Centre, 205 02 Malmö, Sweden; 2 Department of Clinical Science, Center for Diabetes Research, University of Bergen, 5032 Bergen, Norway; 3 Research Unit for General Practice, Danish Aging Research Center, University of Southern Denmark, Odense, Denmark; 4 Steno Diabetes Center Copenhagen, Gentofte, Denmark; 5 Department of Molecular Medicine and Surgery, Karolinska Institute, Stockholm, Sweden; 6 Department of Endocrinology, Metabolism and Diabetes, Karolinska University Hospital, Stockholm, Sweden; 7 Department of Clinical Science and Education, Division of Internal Medicine, Unit for Diabetes Research, Karolinska Institute, South Hospital, Stockholm, Sweden; 8 Center for Diabetes, Academica Specialist Centrum, Stockholm, Sweden; 9 Department of Clinical Sciences, Division of Internal Medicine, Karolinska Institute, Danderyd University Hospital, Stockholm, Sweden; 10 Department of Health Technology, Section for Bioinformatics, Technical University of Denmark, Lyngby, Denmark; 11 Folkhälsan Institute of Genetics, Folkhälsan Research Center, Biomedicum Helsinki, Helsinki, Finland; 12 Abdominal Center, Nephrology, University of Helsinki and Helsinki University Hospital, Biomedicum Helsinki, Helsinki, Finland; 13 Research Programs for Clinical and Molecular Metabolism, Faculty of Medicine, University of Helsinki, Helsinki, Finland; 14 Department of Diabetes, Central Clinical School, Monash University, Melbourne, VIC Australia; 15 Department of Clinical Medicine, University of Copenhagen, Copenhagen, Denmark; 16 Institute for Molecular Medicine Finland FIMM, University of Helsinki, Helsinki, Finland; 17 Department of Public Health and Clinical Medicine, Sunderby Research Unit, Umeå University, Umeå, Sweden; 18 Department of Medicine, University of Gothenburg, Gothenburg, Sweden; 19 Department of Molecular Medicine and Surgery, Rolf Luft Center for Diabetes Research, Karolinska Institutet, Stockholm, Sweden; 20 Karolinska University Hospital, Solna, Stockholm, Sweden; 21 Research Program Unit, Clinical and Molecular Metabolism, University of Helsinki, Helsinki, Finland; 22 Institute of Clinical Physiology, CNR, Pisa, Italy; 23 Centre for Analysis and Synthesis, Department of Chemistry, Lund University, 223 62 Lund, Sweden; 24 Institute of Clinical Medicine, Faculty of Medicine, University of Oslo, Oslo, Norway; 25 Department of Endocrinology, Oslo University Hospital, Oslo, Norway

**Keywords:** Metabolomics, Biomarkers, Diabetes complications, Type 1 diabetes

## Abstract

Identification of biomarkers associated with protection from developing diabetic complications is a prerequisite for an effective prevention and treatment. The aim of the present study was to identify clinical and plasma metabolite markers associated with freedom from vascular complications in people with very long duration of type 1 diabetes (T1D). Individuals with T1D, who despite having longer than 30 years of diabetes duration never developed major macro- or microvascular complications (non-progressors; NP) were compared with those who developed vascular complications within 25 years from diabetes onset (rapid progressors; RP) in the Scandinavian PROLONG (n = 385) and DIALONG (n = 71) cohorts. The DIALONG study also included 75 healthy controls. Plasma metabolites were measured using gas and/or liquid chromatography coupled to mass spectrometry. Lower hepatic fatty liver indices were significant common feature characterized NPs in both studies. Higher insulin sensitivity and residual ß-cell function (C-peptide) were also associated with NPs in PROLONG. Protection from diabetic complications was associated with lower levels of the glycolytic metabolite pyruvate and APOCIII in PROLONG, and with lower levels of thiamine monophosphate and erythritol, a cofactor and intermediate product in the pentose phosphate pathway as well as higher phenylalanine, glycine and serine in DIALONG. Furthermore, T1D individuals showed elevated levels of picolinic acid as compared to the healthy individuals. The present findings suggest a potential beneficial shunting of glycolytic substrates towards the pentose phosphate and one carbon metabolism pathways to promote nucleotide biosynthesis in the liver. These processes might be linked to higher insulin sensitivity and lower liver fat content, and might represent a mechanism for protection from vascular complications in individuals with long-term T1D.

## Introduction

The globally rising incidence and survival of type 1 diabetes (T1D) imposes a considerable socio-economic burden driven by the associated macro- (coronary artery disease, stroke, peripheral artery disease), and microvascular (retinopathy, nephropathy, and neuropathy) complications^1,2^. The Nordic countries have the highest incidence of T1D in the world^3^. Although recent technological advances have improved strategies for insulin delivery, they neither prevent the disease progression nor protect from the chronic diabetic complications^4^. Investigation of protective mechanisms may help to identify new targets for prevention of diabetic complications.

Lack of endogenous insulin due to an autoimmune destruction of the pancreatic ß-cells is a common feature of those with T1D. Chronic hyperglycaemia leads to metabolic adaptations in many cells, aiming to reduce glucose transport and to maintain intracellular glucose concentrations within the physiological range^5,6^. However, some cells and tissues with high energy demands, such as the endothelial cells, ß-cells and neurons, do not adapt their glucose transport efficiently enough, which results in increased intracellular glucose concentrations. In addition, a relative insulin deficiency stimulates gluconeogenesis in the liver and lipolysis in the adipose tissue, resulting in the release of glucose and free fatty acids, and, thereby further increasing the levels of circulating energy sources. A decade ago, Michael Brownlee hypothesized that intracellular energy overload would lead to the overproduction of superoxide in the mitochondria, leading to oxidative stress and predispose to diabetic complications^7^. However, pharmacological interventions targeting these pathways in humans have failed to halt or prevent the development of diabetic complications. Hence, the key question regarding the mechanisms responsible for sustained resistance and protection of organs and cells from damage in individuals with diabetes remains unanswered. The Scandinavian PROLONG (PROtective genetic and non-genetic factors in diabetic complications and LONGevity) initiative in Sweden and Denmark was launched aiming to identify clinical, metabolic and genomic factors promoting protective mechanisms in individuals with long-term T1D who have escaped from the development of diabetic complications. The Norwegian DIALONG study of individuals with long-term T1D was set up to describe undiagnosed coronary artery disease (CAD) and joint stiffness after a duration of diabetes of more than 45 years^8,9^. In the present study, we aimed to characterize clinical and plasma metabolomic features associated with freedom from vascular complications in individuals with long-term T1D in two independent Scandinavian cohorts PROLONG and DIALONG.

## Results

### Clinical characteristics

In total, 482 individuals with long-term T1D were included in the present analyses, of whom 374 were non-progressors (NP) (PROLONG, n = 333; DIALONG, n = 41) and 108 were rapid progressors (RP) (PROLONG, n = 78; DIALONG, n = 30). Descriptive characteristics of NP and RP groups are shown in Tables 1 and 2. Age at onset of the PROLONG participants with T1D occurred in general after puberty (NP and RP, 17.5 ± 9.8 vs. 23.3 ± 14.4, *p* = 0.01) years, and of the DIALONG participants before or during puberty (12.3 ± 6.0 vs. 10.4 ± 6.2 years, *p* = 0.17). Consistently in both cohorts, NPs were characterized by lower fasting plasma triglyceride levels (NP and RP, 0.8 ± 0.3 vs. 1.0 ± 0.5 mmol/L, *p* < 0.05 in PROLONG and 0.7 ± 0.2 vs. 1.0 ± 0.5 mmol/L, *p* < 0.01 in DIALONG), and lower to a lesser degree by lower HbA_1c_ (7.6 ± 1.0 vs. 9.0 ± 1.5%, *p* < 10^–8^ in PROLONG, and 7.3 ± 0.7 vs. 7.7 ± 0.8%, *p* = 0.02 in DIALONG). Reduced fatty liver indices were prominent features in participants free from vascular complications in both studies (fatty liver index (FLI), 0.6 ± 0.7 vs. 0.8 ± 0.8, *p* = 0.01 and 0.5 ± 0.8 vs. 1.5 ± 1.5, *p* < 0.001, and hepatic steatosis index (HSI), 33.7 ± 4.8 vs. 36.0 ± 5.8, *p* = 0.06 and 32.9 ± 3.7 vs. 37.2 ± 4.4, *p* < 0.001; in PROLONG and DIALONG, respectively).

**Table 1:** Clinical characteristics of the PROLONG participants.

Phenotype	Number NP	Number RP	NP	RP	*p* value_a_	*p* value_b_
Age [years]	322	74	58.1 (10.5)	45.1 (14.1)		
Age at diagnosis [years]	322	74	17.5 (9.8)	23.3 (14.4)	0.01*	
T1D duration [years]	333	78	40.5 (8.6)	21.6 (7.9)	< 0.0001	< 0.0001
Sex, male	333	78	144 (43%)	40 (51%)	0.25*	
Smoking, current	311	71	7 (2%)	4 (6%)	0.25*	
Hypertension	331	77	192 (58%)	53 (68%)	0.11*	
Waist [cm]	313	67	51.6 (43.8)	77.4 (34.5)	0.04	0.18
Waist/Hip	313	67	0.9 (0.1)	0.9 (0.1)	0.07	0.19
BMI [kg/m^3^]	324	78	24.8 (3.8)	26.2 (4.7)	0.04	0.12
Systolic BP, sitting [mmHg]	328	76	130.6 (17.5)	126.9 (17.7)	0.19	0.45
Diastolic BP, sitting [mmHg]	328	76	75.5 (9.1)	79.1 (9.1)	0.17	0.27
GAD antibodies [number positive]	311	66	156 (50%)	44 (67%)	0.02	
C-peptide [nmol/L]**	285	62	0.008 (0.003–0.015)	0.003 (0.003–0.014)	0.03	0.02
HbA_1c_ [DCCT]	329	78	7.6 (1.0)	9.0 (1.5)	< 0.0001	
Insulin dosage [units/day]**	218	55	36.5 (29.0–46.0)	57.0 (40.2–72.0)	< 0.001	5.0e−03
eGDR [mg/kg/min]	310	66	7.0 (2.3)	5.7 (2.5)	< 0.0001	< 0.0001
Triglycerides [mmol/L]	319	76	0.8 (0.3)	1.0 (0.5)	< 0.0001	0.02
HDL-C [mmol/L]	319	76	1.8 (0.6)	1.6 (0.6)	0.98	0.24
Cholesterol [mmol/L]	319	76	4.8 (0.8)	4.8 (0.9)	0.63	0.98
LDL-C [mmol/L]	319	76	2.6 (0.7)	2.7 (0.8)	0.91	0.30
eGFR [ml/min/1.73m^3^]	321	74	86.8 (15.6)	91.0 (30.0)	0.57	0.98
ASAT [U/L]	333	76	25.2 (8.9)	21.2 (8.2)	0.01	0.06
ALAT [U/L]	333	78	24.0 (11.7)	21.9 (9.8)	0.01	0.01
ASAT/ALAT	333	76	1.16 (0.42)	1.02 (0.31)	0.80	0.56
GGT [U/L]	332	78	28.2 (43.5)	34.6 (81.7)	0.10	0.33
Fatty liver index (FLI)	297	65	0.60 (0.71)	0.81 (0.77)	0.01	0.05
Hepatic steatosis index (HSI)	324	76	33.7 (4.8)	36.0 (5.8)	0.06	0.39
Retinopathy, n (%)	–	78	–	54 (69%)		
Nephropathy, n (%)	–	78	–	41 (53%)		
CVD, n (%)	–	56	–	2 (3%)		
Lipid treatments	180	57	102 (67%)	29 (51%)	0.54*	
Antihypertensive treatments	180	56	70 (39%)	31 (55%)	0.04*	

Data are mean (sd).

*BP* denotes blood pressure, *NP* non-progressors, *RP* rapid progressors.

Linear regression models: *P*_a_ value = adjusted for center/storage, sex, age, *P*_b_ value adjusted for center/storage, sex, age, HbA_1c_.

*Mann–Whitney test.

**Median (IQR), or count (%).

**Table 2:** Clinical characteristics of the DIALONG participants.

Phenotype	NP	RP	*p* value_a_	*p* value_b_
Age [years]	61.1 (7.1)	61.5 (6.7)		
Age at diagnosis [years]	12.3 (6.0)	10.4 (6.2)	0.17*	
T1D duration [years]	48.8 (3.5)	51.1 (5.2)	0.02	0.07
Sex (male)	16 (39%)	13 (43%)	0.90*	
Smoking, current	2 (5%)	1 (3%)	1.00*	
Hypertension	26 (63%)	16 (53%)	0.54*	
Waist [cm]	85.6 (10.4)	95.0 (13.2)	< 0.001	3.9e−03
Systolic BP [mmHg]	144.8 (19.5)	144.5 (17.2)	0.93	0.79
Diastolic BP [mmHg]	75.7 (8.6)	73.0 (6.6)	0.12	0.31
BMI [kg/m^3^]	24.6 (3.1)	27.4 (3.8)	1.7e−03	0.01
GAD antibodies [kE/L]	0.19 (0.45)	0.17 (0.59)	0.79	0.95
GAD antibodies (positive)	0 (0%)	0 (0%)	n/a	n/a
Anti-insulin	0.39 (0.56)	0.71 (1.0)	0.12	0.15
C-peptide [nmol/L]**	Undetectable	Undetectable	n/a	n/a
HbA_1c_ [DCCT]	7.3 (0.7)	7.7 (0.8)	0.02	
Insulin daily dosage [units/day]**	30.0 (24.0–38.0)	37.0 (34.0–45.8)	0.12	0.16
eGDR [mg/kg/min]	7.2 (1.8)	6.5 (2.2)	0.19	0.28
Triglycerides [mmol/L]	0.7 (0.2)	1.0 (0.5)	4.5e−03	6.7e−03
HDL-C [mmol/L]	2.2 (0.5)	2.0 (0.5)	0.11	0.18
Cholesterol [mmol/L]	5.1 (0.9)	4.9 (1.0)	0.52	0.89
LDL-C [mmol/L]	2.7 (0.8)	2.7 (0.8)	0.88	0.75
CRP [mg/L]	2.2 (2.4)	2.9 (2.9)	0.28	0.54
Creatinine [μmol/L]	69.2 (11.9)	81.2 (28.9)	0.02	0.09
eGFR [ml/min/1.73m^3^]	70.1 (14.4)	64.2 (16.4)	0.16	0.35
ASAT [U/L]	27.3 (8.9)	29.5 (10.7)	0.39	0.44
ALAT [U/L]	24.4 (11.8)	31.1 (14.7)	0.04	0.07
ASAT/ALAT	1.2 (0.3)	1.0 (0.2)	< 0.001	1.3e−03
GGT [U/L]	25.9 (17.7)	37.7 (29.0)	0.04	0.14
Fatty liver index (FLI)	0.5 (0.8)	1.5 (1.5)	< 0.001	4.6e−03
Hepatic steatosis index (HSI)	32.9 (3.7)	37.2 (4.4)	< 0.0001	< 0.001
Retinopathy, n (%)		29 (97%)		
Nephropathy, n (%)		10 (33%)		
CVD, n (%)		12 (40%)		
Statins	14 (34%)	20 (67%)	0.01*	
Beta-blocker	2 (5%)	11 (37%)	2.0e−03*	
ACE/ARB	10 (24%)	21 (70%)	< 0.001*	
Antiplatelet agent	5 (12%)	14 (47%)	3.0e−03*	
Loop diuretics	1 (2%)	7 (23%)	0.02*	

Data are mean (sd).

n_NP_ = 41, n_RP_ = 30.

*BP* denotes blood pressure, *NP* non-progressors, *RP* rapid progressors.

Linear regression models: *P*_a_ value = adjusted for center/storage, sex, age, *P*_b_ value adjusted for center/storage, sex, age, HbA_1c_.

*Mann–Whitney test.

**Median (IQR), or count (%).

GAD antibody positivity at the time of the studies (2011–2015) was less frequent among NPs than RPs (*p* = 0.02) in PROLONG, while there were no differences in DIALONG. Higher estimated glucose disposal rate (eGDR, NP and RP, 7.0 ± 2.3 vs. 5.7 ± 2.5, *p* < 10^–5^), reflecting a better insulin sensitivity, and more residual ß-cell function as assessed by fasting C-peptide (*p* = 0.03) were associated with lack of complications in PROLONG, but not in DIALONG. There was a strong negative correlation between eGDR and liver fat indices (PROLONG: eGDR and FLI ρ = − 0.54, *p* < 2.2e−16 (NP), and ρ = − 0.62, *p* = 3.4E−08 (RP); eGDR vs. HSI ρ = − 0.39, *p* = 1.6e−12 (NP), and ρ = − 0.49, *p* = 4.2E−05 (RP); DIALONG: eGDR and FLI ρ = − 0.47, *p* = 0.002 (NP), and ρ = − 0.66, *p* = 7.0E−05 (RP); eGDR and HSI ρ = − 0.3, *p* = 0.06 (NP), and ρ = − 0.63, *p* = 1.8E−04 (RP)).

Encouraged by the low triglyceride concentrations in the NP group, we also measured the plasma apolipoprotein CIII (APOCIII) levels in the PROLONG cohort, and there were slightly lower APOCIII levels in the NPs as compared to the RPs (*p* = 2.5e−03) (Table 3). The difference remained unchanged and significant after adjustment for HbA_1c_ levels (*p* = 0.02).

**Table 3:** Directly measured metabolites in the PROLONG cohort.

			Mean (SD)			
Metabolite	NP	RP	NP	RP	*p* value_a_	*p* value_b_
APOC3 [mg/dl]	318	59	11.6 (3.9)	12.6 (5.9)	2.5e−03	0.02
Pyruvate [μM]	312	64	95.2 (28.9)	102.5 (27.9)	0.01	0.06
IGF1 [μg/L]	333	78	123.2 (42.3)	150.6 (48.4)	ns	0.1
IGFBP1 [μg/L]	330	75	93.3 (44.5)	72.9 (40.8)	3.6e−03	ns
Glucagon [pg/ml]	312	64	15.3 (12.8)	15.0 (9.6)	ns	ns
Alanine [μM]	313	64	322.0 (58.1)	325.0 (68.0)	ns	ns
Lactate [μM]	311	64	916.1 (380.0)	909.3 (393.3)	ns	ns

Data were winsorized before analyses, and statistical tests performed on log2-transformed data.

*NP* non-progressors, *RP* rapid progressors.

*ns* non-significant (*p* > 0.05).

Linear regression models: *P*_a_ (adjusted for center/storage, sex, age), *P*_b_ (adjusted for center/storage, sex, age, HbA_1c_).

#### Plasma metabolites in NPs and RPs

Univariate results from the metabolomics analyses in the PROLONG and the DIALONG cohorts are reported in the Supplementary Appendix. Untargeted metabolomics analyses in the PROLONG showed higher levels of serine (GC–MS) (*p* = 0.01), phenylalanine (LC–MS) (*p* = 0.01) and taurine (GC–MS) (*p* = 0.01) in the NP group compared to the RP group (Table 4). In addition, using the same GC–MS platform in the DIALONG, lower levels of erythritol, myo-inositol, pyroglutamate and cystine (*p* < 0.05) were observed in the NPs compared to the RPs (Table 4). In the targeted metabolite analyses of DIALONG, higher levels of glycine (GC–MS/MS, *p* = 6.7e−03), serine (GC–MS/MS, *p* = 0.02), phenylalanine (GC–MS/MS, *p* = 0.03), and lower levels of thiamine monophosphate (LC–MS/MS, *p* = 0.02) were associated with NPs as compared to RPs (Table 5), and these differences remained significant after adjustment for HbA_1c_. Furthermore, in the multivariable analyses (Table 6), including HbA_1c_ and fatty liver index, these metabolites were independently associated with NPs, apart from serine which is highly correlated with glycine (r^2^ = 0.47, *p* = 5e−04). We further found lower directly measured plasma pyruvate levels in the NPs compared with the RPs (*p* = 0.01) in PROLONG (Table 3). However, this difference was no more significant after adjustment for HbA_1c_ (*p* = 0.06).

**Table 4:** Significant untargeted metabolites in the cohorts (NP vs. RP).

	PROLONG				DIALONG			
Metabolite	b_a_	b_b_	*p* value_a_	*p* value_b_	b_a_	b_b_	*p* value_a_	*p* value_b_
Serine^1^	0.2	0.1	0.01	ns	0.1	0.1	ns	ns
Phenylalanine^2^	0.3	0.3	0.01	0.03	− 0.2	− 0.1	ns	ns
Carnitine C4^2^	− 0.3	− 0.3	ns	ns	− 0.5	− 0.4	2.4e−03	9.1e−03
Carnitine C5^2^	− 0.1	− 0.1	ns	ns	− 0.6	− 0.6	6.1e−03	0.01
Erythritol^1^	–	–	–	–	− 0.6	− 0.5	4.3e−03	0.03
LPE C20:4^2^	–	–	–	–	− 0.7	− 0.6	9.7e−03	0.03
Pyroglutamate^1^	0	0	ns	ns	− 0.3	− 0.3	0.01	0.03
Myo-inositol^1^	− 0.1	− 0.1	ns	ns	− 0.4	− 0.3	0.02	ns
Glutamate^1^	− 0.2	0	ns	ns	− 0.7	− 0.6	0.02	ns
Hydroxyproline^1^	− 0.2	− 0.1	ns	ns	− 0.5	− 0.4	0.02	0.05
Cystine^3,1^	− 0.1	− 0.1	ns	ns	− 0.6	− 0.5	0.02	0.03
Ornithine^3,1^	− 0.1	− 0.1	ns	ns	− 0.5	− 0.4	0.02	ns
Hippuric acid^2^	–	–	–	–	− 0.6	− 0.5	0.02	ns
Carnitine^2^	–	–	–	–	− 0.1	− 0.1	0.03	ns
Cystine^4,1^	− 0.2	− 0.2	ns	ns	− 0.8	− 0.8	0.03	0.05
Creatinine^1^	− 0.1	− 0.1	ns	ns	− 0.5	− 0.4	0.03	ns

Data were winsorized before analyses, and statistical tests performed on log2-transformed data.

*ns* non-significant (*p* > 0.05).

*NP* non-progressors, *RP* rapid progressors.

n_NP_ = 226, n_RP_ = 45 for PROLONG, ^1^n_NP_ = 39, n_RP_ = 30: ^2^n_NP_ = 40, n_RP_ = 30 for DIALONG.

Linear regression models: *P*_a_ (adjusted for sex, age), *P*_b_ (adjusted for sex, age, HbA_1c_), both models are adjusted for center/storage for PROLONG, b for effect size of binary group coefficient.

^1^GC-MS, ^2^UHPLC.

**Table 5:** Top associated targeted metabolites in the DIALONG study.

	Mean (SD)			
Metabolite	NP	RP	*p* value_a_	*p* value_b_
Glycine [μmol/L]^1^	314.4 (62.2)	274.6 (45.8)	6.7e−03	8.7e−03
Serine [μmol/L]^1^	127.1 (26.7)	112.4 (28.3)	0.02	0.03
Phenylalanine [μmol/L]^1^	62.6 (7.2)	58.0 (7.7)	0.03	0.04
Tot. thiamine* [nmol/L]^2^	10.5 (6.4)	15.1 (12.2)	0.03	0.04

Data were winsorized before analyses, and statistical tests performed on log2-transformed data.

*NP* non-progressors, *RP* rapid progressors. n_NP_ = 25, n_RP_ = 27.

Linear regression models: *P*_a_ (adjusted for sex, age), *P*_b_ (adjusted for sex, age, HbA_1c_).

^1^Bevital GC–MS/MS, ^2^Bevital LC–MS/MS.

*Tot. thiamine is calculated as total of thiamine monophosphate and thiamine.

**Table 6:** Multivariate analysis with top associated targeted metabolites in the DIALONG study.

Metabolite	b_a_	*p* value_a_	b_b_	*p* value_b_
Glycine^1^	6.6	0.01	7.6	0.01
Serine^1^	− 0.1	0.95	0.6	0.73
Phenylalanine^1^	11.5	0.01	15.0	0.01
Thiamine monophosphate	− 4.7	0.01	− 5.5	0.01

Correlation between Serine and Glycine is 0.46 in all (*p* = 0.0005), 0.51 in RPs (*p* = 0.007) and 0.3 in NPs (ns).

Data were winsorized before analyses, statistical tests performed on log2-transformed data.

*NP* non-progressors, *RP* rapid progressors. n_NP_ = 25, n_RP_ = 27.

Linear regression models: *P*_a_ (adjusted for sex, age, FLI), *P*_b_ (adjusted for sex, age, FLI, HbA_1c_).

^1^Bevital GC–MS/MS, ^2^Bevital LC–MS/MS.

### Comparison of NPs and RPs with healthy controls

Individuals with long-term T1D in DIALONG showed established features of increased lipolysis, including elevated levels of plasma glycerol (*p* = 0.004), long-chain free fatty acids (*p* ≤ 0.01) and ß-hydroxybutyrate (*p* = 1.8e−06) (Supplementary Table 5) as compared to the healthy individuals. Significantly elevated levels of picolinic acid (*p* = 6.8e−06) were found in those with T1D as compared to the healthy controls by using targeted metabolite profiling (Table 7). Individuals with long-term T1D who never developed major complications (NPs) had higher plasma levels of serine (*p* = 0.04) and reduced fatty liver index (*p* = 6.0e−03, and after adjustment for HbA_1c_ *p* = 8.7e−03) as compared to the healthy age-matched individuals (Table 7 and Supplementary Table 4).

**Table 7:** Top associated targeted metabolites in the cohorts (patients with T1D vs. controls).

	Control	T1D (NP + RP)		NP		RP	
Metabolite	Mean (sd)	Mean (sd)	*p* value (*p*_a_/*p*_b_)	Mean (sd)	*p* value (*p*_a_/*p*_b_)	Mean (sd)	*p* value (*p*_a_/*p*_b_)
Picolinic acid (nmol/L)^2^	35.4 (12.1)	53.2 (20.7)	< 0.0001/ < 0.001	57.0 (19.7)	< 0.0001/ < 0.001	49.7 (21.2)	1.2e−03/0.05
3-Hydroxyisobutyrate (μmol/L)^1^	17.0 (3.6)	21.4 (6.1)	< 0.001/ < 0.001	22.0 (6.0)	3.8e−04/0.01	20.8 (6.2)	7.9e−03/0.02
β-Hydroxybutyrate (μmol/L)^1^	60.0 (51.3)	212.6 (224.3)	< 0.0001/ < 0.001	202.3 (255.0)	3.1e−04/0.03	222.2 (196.2)	< 0.0001/ < 0.001
Nicotinamide (nmol/L)^2^	261.0 (92.1)	211.2 (61.2)	8.0e−03/ns	207.7 (50.0)	0.02/ns	214.4 (71.0)	0.04/ns
N1-methylnicotinamide (nmol/L)^2^	151.7 (76.3)	119.6 (71.2)	0.03/ns	108.5 (63.5)	8.2e−03/ns	129.9 (77.4)	ns/ns
Serine (μmol/L)^1^	114.0 (17.0)	120.6 (28.0)	ns/0.02	128.0 (26.7)	0.02/0.04	113.7 (27.7)	ns/ns
Valine (μmol/L)^1^	242.4 (32.6)	256.9 (42.8)	ns/2.0e−03	262.2 (46.6)	ns/0.04	252.0 (39.1)	ns/0.02
Leucine (μmol/L)^1^	118.3 (19.0)	125.0 (23.2)	ns/8.6e−03	126.3 (23.5)	ns/0.03	123.7 (23.3)	ns/ns
Methionine (μmol/L)^1^	27.7 (2.9)	28.7 (5.2)	ns/0.05	30.2 (5.6)	ns/0.01	27.3 (4.5)	ns/ns
Tot. homocysteine (μmol/L)^1^	11.2 (2.1)	10.9 (3.2)	ns/0.03	10.5 (2.2)	ns/ns	11.2 (3.9)	ns/0.02
Quinolinic acid (nmol/L)^2^	375.2 (90.2)	375.5 (156.2)	ns/0.01	350.3 (124.8)	ns/ns	398.8 (179.6)	ns/1.1e−03
Methylmalonic acid (μmol/L)^1^	1.2 (0.04)	1.2 (0.05)	ns/ns	1.2 (0.06)	ns/ns	1.2 (0.05)	ns/8.7e−03
Histidine (μmol/L)^1^	73.6 (7.0)	76.6 (7.5)	ns/ns	75.0 (7.5)	ns/ns	72.2 (6.3)	0.02/ns
Glycine (μmol/L)^1^	287.5 (80.7)	294.1 (55.2)	ns/ns	314.1 (58.3)	0.02/ns	275.6 (45.8)	ns/ns
Phenylalanine (μmol/L)^1^	62.9 (8.0)	61.2 (7.7)	ns/ns	63.6 (7.1)	ns/0.03	58.9 (7.6)	ns/ns
Kynurenine (μmol/L)^1^	2.5 (0.3)	2.5 (0.4)	ns/ns	2.5 (0.3)	ns/ns	2.5 (0.4)	ns/7.0e−03
3-Hydroxykynurenine (nmol/L)^2^	42.4 (11.8)	48.8 (18.9)	ns/ns	46.0 (13.4)	ns/ns	51.3 (22.9)	ns/0.04

Data were winsorized before analyses, and statistical tests performed on log2-transformed data.

*ns* non-significant (*p* > 0.05), *NP* non-progressors, *RP* rapid progressors. n_control_ = 27, n_NP_ = 25, n_RP_ = 27.

Linear regression models: P_a_ (adjusted for sex, age), P_b_ (adjusted for sex, age, HbA_1c_).

^1^Bevital GC–MS/MS, ^2^Bevital LC–MS/MS.

## Discussion

One of the main findings in the present observational study was markedly lower fatty liver index in both studies, and a significantly higher insulin sensitivity index in the PROLONG NPs as compared to RP patients. Recently, there have been numerous reports on an alarming increase in the prevalence of non-alcoholic fatty liver disease (NAFLD), reaching an estimate of almost 70% of individuals with T1D, but also in non-diabetic individuals^10^. Accumulation of fat in the liver is associated with a four- to fivefold increased risk of macro- and microvascular complications^11–13^. With the increasing rates of obesity, this may be further accompanied by increased insulin resistance, a phenomenon termed as “double diabetes”, which is characterised by impaired action of endogenous or exogenous insulin in the target tissues in individuals with T1D in a similar fashion as described in T2D^14^.

Key components of the fatty liver index—triglycerides and waist circumference—are well established markers of insulin resistance. Our finding of elevated triglycerides in the RPs is in line with the Joslin Medalist and the Golden Years studies, which reported higher triglyceride levels in individuals with long-standing T1D and macroalbuminuria^15,16^. Also, the FinnDiane study reported similar features^17^. Both the Golden Years and the Joslin Medalist studies proposed that elevated HDL cholesterol protects against cardiovascular diseases. In our cohorts, the differences in HDL cholesterol between NP and RP did not reach statistical significance, possibly because our RP groups included mostly patients with microvascular complications where HDL seemingly plays less of a role. Another explanation for the lack of association may be an absence of protective properties of HDL at the high levels^18^. Therefore, the interpretation could be specifically challenging in T1D, in which HDL particles could also be atherogenic, if HDL is high. Lower levels of triglycerides in the NP group are compatible with an increased suppression of lipolysis as supported by findings of reduced APOCIII levels in the NPs; importantly, adjustment for HbA_1c_ did not change this result, confirming higher insulin sensitivity and stronger inhibition of lipolysis despite lower insulin dosage in NPs. These findings support a recent report demonstrating that serum APOCIII levels predict incident coronary artery disease independently from diabetes duration and HbA1c in individuals with T1D.

Another correlate of fatty liver index is gamma-glutamyltransferase (GGT), a membrane-bound enzyme, which is indicative of cell damage when elevated in the circulation and found significantly lower in the DIALONG NPs. GGT plays a crucial role in regulation of the redox balance by metabolizing extracellular reduced glutathione to provide the amino acid cysteine for de novo synthesis of glutathione^19^. Interestingly, we also observed high plasma levels of the non-essential amino acids, serine and glycine in the NP group, which were also negatively associated with fatty liver indices in the NP group (Figure S1). Glycine serves as an intermediate in the glutathione biosynthesis, although levels of this amino acid are not rate-limiting, whereas serine is a substrate for synthesis of both cysteine and glycine in the glutathione backbone. Reduced levels of serine have previously been linked to NAFLD in individuals with T2D^20^, while elevated serine levels is also a common feature associated with cancer^21^. Serine is also an intermediate in sphingolipid synthesis. Lower serine levels in RP may suggest increased incorporation of this amino acid into lipids, including ceramides and other compounds that have been shown to accumulate in NAFLD and insulin resistance^22^. The NP group had also higher serine levels compared with the control group without diabetes. Moreover, glycine and serine are biosynthetically linked, and serine can be converted to glycine in the folate cycle, which, in turn, fuels carbons for nucleotide synthesis. The folate cycle is depending on the pentose phosphate pathway (PPP), which plays a key role in supplying intermediates for nucleotide synthesis and reducing equivalents to control the redox balance^23^.

A key metabolite that can help to reduce oxidative stress by electron donation is NADPH, which is largely generated by the PPP in cells with high energy demands, such as the hepatocytes and the erythrocytes. Generation of precursors (glyceraldehyde 3-phosphate, ribose-5-phosphate and erythrose 4-phosphate) for nucleotide biosynthesis is a main function of PPP. In situations of intracellular fuel overload, shunting glycolytic intermediates into the PPP yields a relative energy loss in combination with an improved antioxidant and restorative capacity, via elevated cellular NADPH levels^24^.

In support of an active PPP, in the NPs we observed lower levels of the vitamin B1 (thiamine) derivative, which potentially could indicate an elevated utilization of thiamine in the NPs. Thiamine is an essential cofactor of the PPP, which generates NADPH and other phosphates by translocases. Reduced levels of thiamine have previously been associated with an increased risk of complications in individuals with T1D and T2D^25,26^. Thiamine deficiency is common in diabetic patients and supplementation with benfotiamine, a synthetic thiamine activator, has been shown to prevent the development of diabetic neuropathy, nephropathy and retinopathy in experimental diabetic animals^27,28^. However, supporting human data are still lacking^29^. Beneficial effects of thiamine monophosphate administration have also been demonstrated in a number of neurological disorders including Alzheimer disease and epilepsy^30^.

Additionally, we observed higher levels of the aromatic amino acid phenylalanine in NP patients. There is accumulating evidence showing a link between branched-chain and aromatic amino acids including valine, leucine, isoleucine, phenylalanine, and tyrosine with insulin resistance and T2D risk^31^. The association of elevated phenylalanine levels with relative protection from complications observed in our study might hence seem to be somewhat discordant with these findings. The fact that valine and leucine levels did not differ in NPs vs. RPs could suggest that phenylalanine might play a unique role in the metabolism that is different from what is seen in insulin resistance. In this context, the question whether generated in the PPP erythrose-4-phosphate is preferentially utilized as a precursor in the synthesis of the amino acid phenylalanine. This is supported by the lower levels of erythritol in the DIALONG NPs, which is recently shown^32^, is produced in the PPP by reduction of erythrose-4-phosphate to erythritol-4-phosphate and further released in the form of erythritol. Notably, elevated levels of erythritol have been reported to be associated with progression to T2D^33^, development of retinopathy in T2D^34^ and progression to microalbuminuria in T1D^35^.

We observed decreased pyruvate in the NP group, which could support the shift of glycolysis towards the PPP. Phenylalanine and serine have been ascribed a role as allosteric inhibitors and activators, respectively, of pyruvate kinase^36^. This is in line with the Joslin Medalist study showing upregulation of pyruvate kinase in the kidney biopsies from individuals with more than 50 years of T1D without kidney disease^37^. Recently, proteomics analyses of plasma identified enrichment of biomarkers from the glycolytic pathways, pentose phosphate- and pyruvate metabolism in the T1D Medallists that were protected from chronic kidney disease^38^. Pyruvate kinase is activated by insulin, and whether phenylalanine and serine may counteract the insulin deficiency-related reduction in glycolytic flux remains to be established. Given that there were no differences in the levels of lactate and alanine between NPs and RPs, which would be expected in the case of increased glycolytic flux, another possible explanation for the reduced pyruvate levels could be its utilization in one carbon metabolism—another essential pathway regulating nucleotide biosynthesis.

In addition, in the DIALONG T1D cohort we found elevated levels of picolinic acid, which is produced from tryptophan in the kynurenine pathway. These results support recent findings of an HbA_1c_-independent elevation of the kynurenine pathway intermediates in the urine of children with T1D^39^. The authors suggested that the kynurenine pathway could be responsible for the neuronal damage observed in T1D children and young adults^40^. The cytoprotective effects of the kynurenine pathway has been linked to neuronal DNA repair^41^. Complete tryptophan oxidation results in the *de-novo* synthesis of the intracellular pyrimidine—nicotinamide adenine dinucleotide (NAD^+^). NAD^+^ serves as a rate limiting substrate for the master sensors of energy metabolism, sirtuins (SIRTs), and is responsible for the DNA repair, Poly (ADP-ribose) polymerases (PARPs)^42^. During recent years, boosting of NAD + levels in vivo has been suggested as a means to combat a number of age-related disorders and to prolong healthy lifespan^43^. Moreover, we found lower levels of NAD + precursors, nicotinamide and methylnicotinamide, in individuals with T1D, as compared to healthy controls. Hence, these data may suggest an alteration in the branching point of the kynurenine pathway, which either buds off into synthesis of the neuroprotective picolinic acid, or the neurotoxic quinolinic acid, which is later metabolized to nicotinamide. Notably, the neuroprotective effects of picolinic acid is believed to result from the ability of this metabolite to chelate iron and zinc^44^, thereby antagonizing the cytotoxic quinolinic acid.

Our data of significantly higher insulin sensitivity, as estimated by the eGDR-index, in the PROLONG NPs support the idea of higher synthesis of nucleotides in the liver described to be controlled by insulin^45^. A number of studies in healthy^46,47^ and T1D^48^ individuals suggested that fatty liver indices may serve as surrogate markers for abnormal insulin sensitivity. The present findings of a strong inverse correlation between insulin sensitivity and liver fat indices are in line with a recent study from the FinnDiane Study Group reporting that obese long-term T1D with relatively few complications had higher insulin sensitivity of hepatic glucose production and insulin sensitivity to lipolysis, and lower liver fat content measured with proton magnetic resonance spectroscopy as compared to age-matched healthy individuals^49^.

We did not detect differences in insulin sensitivity between NP and RP in the DIALONG study. One explanation, however, could be a selection bias for survival in our study as RPs in the DIALONG were older, had longer diabetes duration with lower HbA_1c_ and lower prevalence of hypertension. Better insulin sensitivity in long-term DIALONG RPs would be in agreement with the recent report from the Swedish National Diabetes Registry showing gradually increased risk of CVD-related death with declined eGDR^50^. Nevertheless, the DIALONG NPs demonstrated better insulin sensitivity for the given level of liver fat as compared to RPs or healthy individuals, supporting an overall better insulin sensitivity in NPs.

Finally, we detected nearly twofold higher levels of residual C-peptide in PROLONG NPs, which could be a plausible explanation for the protection against complications^51^. This, however, was not replicated in the DIALONG study, which included individuals with an earlier age of T1D onset, and absence of endogenous insulin production.

### Limitations

Although PROLONG and DIALONG represent large studies of individuals with long-term T1D who never progressed to diabetic complications, the sample size is still limited. Metabolomics analyses were conducted using two analytical platforms, of which targeted metabolomics clearly represents the most reliable assessment of metabolite quantities. Replication in independent cohorts including entire population of people with T1D and information on vascular complication status using the same platform is an important next step.

### Future perspectives

Present findings highlight importance of future analyses of biomarkers linking liver metabolism and vascular complications in diabetes. Such analyses may include, for example, the possible role of serological biomarkers of endothelial dysfunction such as Endocan Serum Levels in Patients with Non-Alcoholic Fatty Liver Disease with or without Type 2 Diabetes^52^.

**Figure 1:**
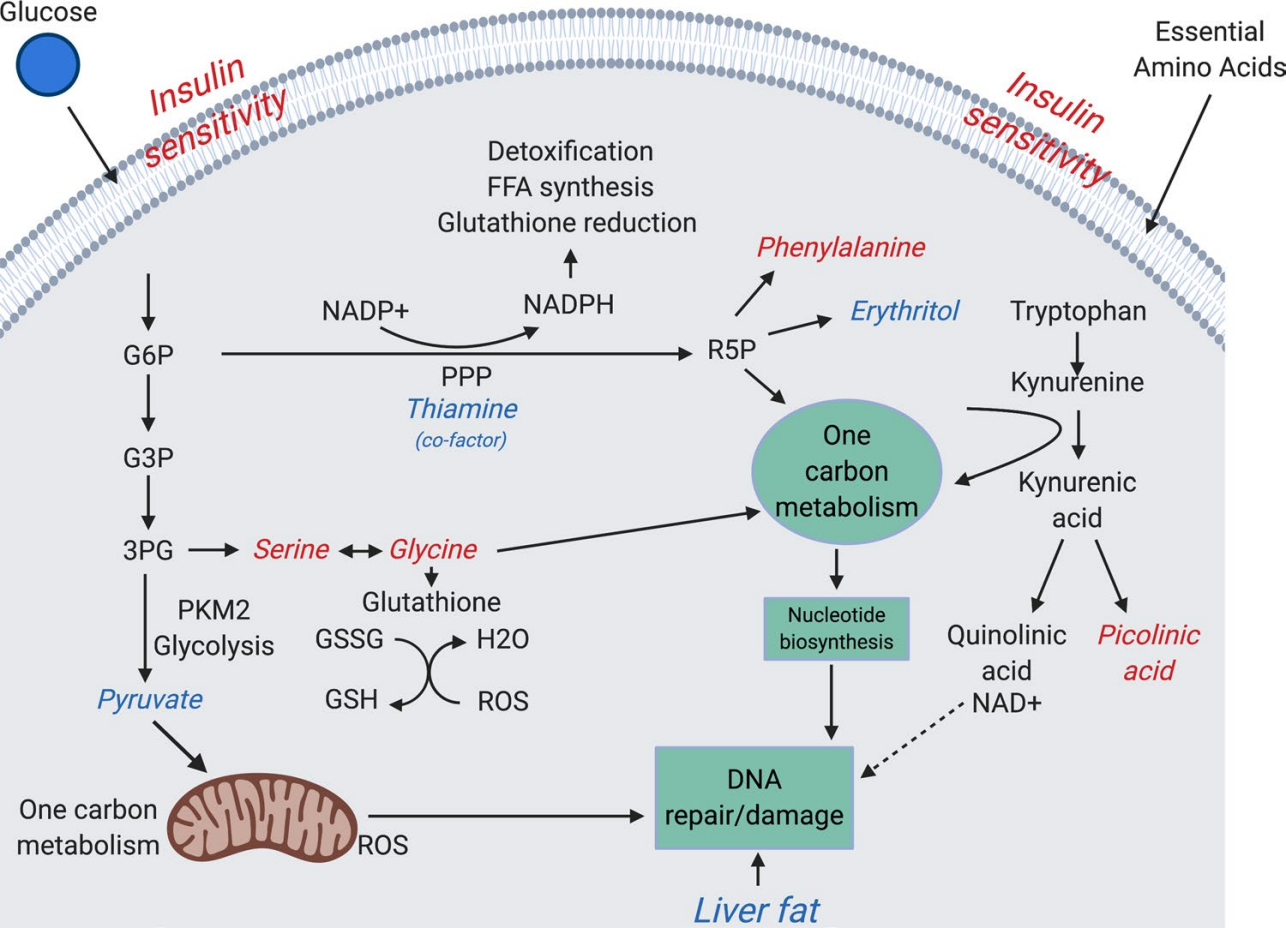
Schematic representation of the proposed mechanisms underlying protection from disease progression to vascular complications in patients with long-standing T1D. Higher insulin sensitivity and lower glucose levels (measured with HbA_1c_), and also lower accumulation of fat in the liver characterize patients with long-term T1D who never progress to vascular complications (NPs). In the liver cell, glycolytic intermediates are actively shunted into the pentose-phosphate pathway (PPP) as indicated by reduced essential cofactor thiamine, increased phenylalanine and reduced erythritol. Generated NADPH is used in reductive biosynthesis of glutathione and detoxification processes to maintain antioxidant capacity. Ribose-5-phosphate (R5P) generated in PPP, and serine, glycine and pyruvate generated in glycolysis are used in the one carbon metabolism and nucleotide biosynthesis to improve restorative capacity. Metabolism of essential amino acid tryptophan generates protective picolinic acid. Elevated factors are in red, reduced are in blue (Created with BioRender.com).

In conclusion, our data suggest that shunting of glycolytic substrates in the liver towards the PPP and one carbon metabolism to promote nucleotide synthesis is potentially beneficial for the protection against vascular complications in individuals with long-term T1D as schematised in Fig. 1. These processes might be linked to better insulin sensitivity and lower fat accumulation in the liver. Further studies investigating cellular levels of the metabolites and their genomic regulation by insulin are needed.

## Methods

### Study participants

Information on the PROLONG eligibility criteria and complication status was based on initial electronic hospital records^53^. Recruitment of the participants was initiated at the Scania University Hospital, Malmö, Sweden, in February 2011. The recruitment continued at the Karolinska University Hospital, Danderyd Hospital and South Hospital in Stockholm, Sunderby Hospital in Luleå, University Hospital of Umeå, Sahlgrenska University Hospital in Gothenburg, all in Sweden, and the Steno Diabetes Center Copenhagen (SDCC), Gentofte, Denmark. The last PROLONG study visits were conducted at the SDCC in 2015. Individuals with T1D were classified based on their diabetes complication status. Non-progressors (NPs) were defined as those with diabetes duration of more than 30 years and who did not develop any major complications (diabetic nephropathy, proliferative retinopathy, myocardial infarction, stroke or chronic foot ulcer), whereas rapid progressors (RPs) were defined as those, who developed any of the above-mentioned complications within 25 years of diabetes duration. NPs who prior to or at the PROLONG study visit were diagnosed with any of the above-mentioned complications were designated “late-progressors” and excluded from the present analyses.

DIALONG is a Norwegian study of individuals with long-term T1D with a disease duration of more than 45 years (n = 103) and age-matched healthy controls (n = 75). The DIALONG study aims to investigate the prevalence of and risk factors for CAD and joint stiffness in older T1D individuals. The design and recruitment protocols for the DIALONG study have been published in detail previously^8^. Both the PROLONG and the DIALONG studies were approved by the local ethics committees (PROLONG-Sweden, Regional Ethics Review Board, Department 1, Lund, Sweden, Dnr 777/2009, PROLONG-Denmark, The Capital Region Ethics Committee, Hillerød, Denmark, Dnr H-2-2013-073, DIALONG, South-East regional health authority, panel D, Norway, 2014/851; 2019/28,968, for data analysis of both studies in UiB, West regional health authority, Norway, REK-2019/1,324) and conducted in accordance with local institutional and national regulations.

#### Definition of diabetic complications

Diabetic nephropathy was defined as the presence of (1) macroalbuminurea ≥ 200 µg/min in a timed overnight urine collection, (2) an albumin/creatinine ratio > 300 mg/g as macroalbuminurea, or < 300 mg/g or > 30 mg/g as microalbuminuria in at least two out of three consecutive urine collections/morning urine samples (PROLONG-Denmark, DIALONG) or based on a documented diagnosis of diabetic kidney disease (PROLONG-Sweden). Proliferative diabetic retinopathy was assessed with fundus photography and defined as the presence of proliferative retinopathy in at least one eye and/or laser therapy (panretinal photocoagulation), or non-traumatic blindness. CVD was defined as non-fatal myocardial infarction and/or stroke (haemorrhagic or ischemic) (PROLONG-Sweden, PROLONG-Denmark) and balloon angioplasty or coronary artery bypass surgery (DIALONG).

### Procedures and measurements

In the PROLONG study, participants fasted overnight (> 8 h) and were instructed not to take their medication in the morning of the visit. Trained diabetes research nurses and biomedical analysts performed the physical examination following standard operating procedures. On the day of the examination, a signed informed consent was obtained, and blood and urine samples taken. The participants were also asked to fill out a detailed questionnaire. These self-reported records were then validated by the diabetes nurse at the clinical research visit and this information was stored in a database at the Scania University Hospital, Malmö, Sweden, and at the Trial Partner Data Management, Region Midtjylland in Denmark.

#### Questionnaires

Questionnaires were mailed to each participant prior to the clinical visit. At the SDCC, the questionnaire was also accessible electronically. The questionnaire included sections regarding personal medical history, complication status, family history of diabetes, education, physical activity, diet, smoking, psychosocial health and social status. Female participants were asked for details concerning pregnancy. Questionnaires were controlled by the research nurse, and the data were checked for outliers, nonsense values and cleaned before uploaded to the study database. In Sweden, all data obtained by questionnaires was transferred to the database at the Scania University Hospital in Malmö for central error detection. In Denmark, questionnaire data was verified at SDCC and stored at Trial Partner Data Management, Region Midtjylland.

#### Anthropometric measurements

Height was measured to the nearest millimetre. Weight was measured in light indoor clothing, without shoes, to the nearest 0.1 kg. Clothes were estimated to weigh 0.5 kg, which was deducted from the total weight. Body mass index (BMI) was calculated according to the formula weight (kg)/height^2^ (m). Waist and hip circumferences were measured at the mid-point between the lower costal margins and the level of the anterior superior iliac crest to the nearest millimetre with the participant in a relaxed, standing position using standard measuring tape. Systolic and diastolic blood pressure was measured using calibrated equipment. The patient was placed in a chair and after 10 min rest, blood pressure was measured using the mean of two measurements.

#### Biochemical measurements

The blood samples were analysed at the clinical chemistry laboratories at the participating centres. Serum C-peptide and plasma GAD65 auto-antibodies (GAD ab) were measured by electrochemiluminescence-immunoassay/ECLIA and immunometric ELISA, respectively, at the Department of Clinical Chemistry in Malmö, Sweden. Serum IGF-1 was determined by RIA after separation of IGFs from insulin-like growth factor binding proteins (IGFBPs) by acid ethanol extraction and cryoprecipitation. To minimize interference of remaining IGFBPs a truncated form of IGF-1 [des(1–3) IGF-1] was used as radioligand^54^, with intra- and inter-assays coefficient of variation of 4% and 11%, respectively. Serum IGFBP-1 concentrations were determined by RIA^55^, with intra- and inter assays coefficients of variation of 3% and 10%, respectively. Creatinine was measured using enzymatic, colorimetric methods and albumin using immunoturbidimetric methods in the first morning urine sample to calculate the albumin-creatinine ratio (ACR). Urine and plasma samples were stored at − 80 °C for further analyses. Insulin dosage was self-reported and the cumulative daily insulin doses were calculated based on the self-reported questionnaire.

#### Estimated metabolic parameters

The albumin/creatinine ratio was calculated using the formula: (U-albumin mg/l × 8.84)/(U-creatinine (μmol/l)/1,000). eGFR was calculated using the following CKD-EPI formula: eGFR = 186 × serum creatinine^−1.154^ × age^−0.203 ^× (0.742 if female). VLDL cholesterol (VLDL-C) was calculated using the formula: VLDL-C = triglycerides (mmol/l)/2.2, if the triglyceride values were ≤ 5.05 mmol/l. LDL cholesterol was calculated using the Friedewald’s equation (LDL-C = TC–VLDL-C–HDL-C mmol/l), and the estimated glucose disposal rate (eGDR) based on the formula eGDR = 21.158 + (− 0.09 × waist [cm]) + (−3.407 × hypertension [yes = 1/no = 0]) + (− 0.551 × HbA_1c_ [%]), where hypertension was defined as a systolic blood pressure > 140 mmHg or a diastolic blood pressure > 90 mmHg or the use of antihypertensive medication^56^. Hepatic steatosis index (HSI) was calculated using the formula: HSI = 8 × (ALAT/ASAT) + BMI + (2 if female)^57^, and fatty liver index (FLI) was defined as FLI = 100 × *log* [0.953 × ln(triglycerides) + 0.139 × BMI + 0.718 × ln(GGT) + 0.053 × waist – 15.745], where *log(x)* = 1/1 + e^−*x*^^58^. For the DIALONG study these procedures have been published previously^8^.

### Metabolomics

Plasma metabolites were determined using gas chromatography/time-of-flight mass spectrometry (GC/TOF–MS) (LECO Pegasus III TOF electron impact MS, LECO Corp., St. Joseph, MI)^59,60^ and reverse-phase ultra-high performance liquid chromatography/quadrupole time-of-flight mass spectrometry (UHPLC/QTOF-MS) (1,290 Infinity UPLC/6,550 iFunnel Q-TOF, Agilent Technologies, Santa Clara, CA) (DIALONG and PROLONG) in both positive and negative electrospray ionization mode^61^. Variation within each batch was corrected by using stable isotope labelled internal standards^62^. Samples or metabolites with more than 30% missing values (n = 8) were removed. The PROLONG samples were split into three batches, with the first two batches including samples from the Swedish participants and the third batch only samples from the SDCC. It I of note that the SDCC samples for GC were run 7 months after the Swedish samples (2015), while SDCC samples for UHPLC were run a year after Swedish samples (2015 and 2016). The UHPLC method was run over 6 months after GC, with the exact time difference dependent on the batch. However, data were adjusted for batch effects (between-day analysis) using the COMBAT algorithm^63^. In addition, targeted metabolomics on the DIALONG cohort was performed at Bevital (www.bevital.no) using GC–MS/MS (panel B) and LC–MS/MS (panel D).

### Statistical analyses

All raw data were inspected for quality and distribution, and the outliers were removed using the MAD-method^64^ adjusted for non-symmetric distributions. All metabolite data and non-normally distributed clinical variables were log2-transformed before analysis. Data used in correlation analyses were mean-centred and log2-transformed. Clinical categorical data were compared using Pearson's chi-square test. The group medians were compared using Mann–Whitney U-test. Linear regression models were used to study the association of metabolites and complication group status corrected for covariates: age, sex, recruiting centre, year of visit, and HbA_1c_ where appropriate. All analyses were performed using R, version 3.4.4 and relevant packages. Statistical tests were two-sided with a significance level of *p* < 0.05.

## Supplementary information

Supplementary file1 (DOCX 171 kb)
