# Supplementary material for: Liver nucleotide biosynthesis is linked to protection from vascular complications in individuals with long-term type 1 diabetes
Source: Sci Rep. 2020 Jul 14;10:11561. doi: 10.1038/s41598-020-68130-y (PMC7360755; doi:10.1038/s41598-020-68130-y)
Supplement: Supplementary file 1 — Supplementary file1 (DOCX 171 kb) [file 41598_2020_68130_MOESM1_ESM.docx]

**Liver nucleotide biosynthesis is linked to protection from vascular complications in individuals with long-term type 1 diabetes**

Ruchi Jain^1^, Türküler Özgümüş^2^, Troels Mygind Jensen^3,4^, Elsa du Plessis^2^, Magdalena Keindl^2^, Cathrine Laustrup Møller^4^, Henrik Falhammar^5,6^, Thomas Nyström^7^, Sergiu-Bogdan Catrina^5,6,8^, Gun Jörneskog^9^, Leon Eyrich Jessen^10^, Carol Forsblom^11,12,13^, Jani K Haukka^11,12,13^, Per-Henrik Groop^11,12,13,14^, Peter Rossing^4^, Leif Groop^1,15^, Mats Eliasson^16^, Björn Eliasson^17^, Kerstin Brismar^6^, Mahmoud Al-Majdoub^1^, Peter M. Nilsson^1^, Marja-Riitta Taskinen^18^, Ele Ferrannini^19^, Peter Spegel^1^, Tore Julsrud Berg^20^, Valeriya Lyssenko PhD ^1,2,*^

^1^Dept. of Clinical Science, Lund University Diabetes Centre, Malmö, Sweden.

^2^Dept. of Clinical Science, Center for Diabetes Research, University of Bergen, Bergen, Norway.

^3^University of Southern Denmark

^4^Steno Diabetes Center Copenhagen, Gentofte, Denmark.

^5^Dept. of Molecular Medicine and Surgery, Karolinska Institute, Stockholm, Sweden.

^6^Dept. of Endocrinology, Metabolism and Diabetes, Karolinska University Hospital, Stockholm, Sweden.

^7^Dept. of Clinical Science and Education, Division of Internal Medicine, Unit for Diabetes Research, Karolinska Institute, South Hospital, Stockholm, Sweden.

^8^Center for Diabetes, Academica Specialist Centrum, Stockholm, Sweden

^9^Karolinska Institute, Department of Clinical Sciences, Danderyd University Hospital, Division of Internal medicine, Stockholm.

^10^Dept. of Health Technology, Section for Bioinformatics, Technical University of Denmark, Lyngby, Denmark

^11^Folkhälsan Institute of Genetics, Folkhälsan Research Center, Biomedicum Helsinki, Finland.

^12^Abdominal Center, Nephrology, University of Helsinki and Helsinki University Hospital, Biomedicum Helsinki, Finland.

^13^Research Programs for Clinical and Molecular Metabolism, Faculty of Medicine, University of Helsinki, Helsinki, Finland

^14^Department of Diabetes, Central Clinical School, Monash University, Melbourne, Victoria, Australia

^15^Institute for Molecular Medicine Finland FIMM, University of Helsinki, Finland,

^16^Dept. of Public Health and Clinical Medicine, Sunderby Research Unit, Umeå University, Sweden.

^17^Department of medicine, University of Gothenburg, Gothenburg, Sweden.

^18^Research Program Unit, Clinical and Molecular Metabolism, University of Helsinki, Helsinki, Finland.

^19^Department of Clinical and Experimental Medicine, University of Pisa School of Medicine, Pisa, Italy.

^20^Institute of Clinical Medicine, Faculty of Medicine, University of Oslo, Oslo, Norway

^*^Corresponding author

Valeriya Lyssenko MD, PhD,

Department of Clinical Science, University of Bergen, 5032, Bergen, Norway. TEL: +47-55-975269; E-mail: [valeriya.lyssenko@uib.no](mailto:valeriya.lyssenko@uib.no)

Department of Clinical Sciences/Diabetes & Endocrinology, Lund University, 205 02 Malmö, Sweden. E-mail: [valeriya.lyssenko@med.lu.se](mailto:valeriya.lyssenko@med.lu.se)

**Supplementary Appendix**

**Table S1. Associations of untargeted metabolites in the PROLONG study (NP vs RP groups).**

| **GC-MS** | p_mw_ | p_A_ | b_A_ | p_B_ | b_B_ | p_C_ | b_C_ |
| --- | --- | --- | --- | --- | --- | --- | --- |
| α-Hydroxybutyrate | 0.26 | 0.97 | 0 | 0.65 | 0.1 | 0.7 | 0.1 |
| β-Hydroxybutyrate | 0.05 | 0.46 | 0.2 | 0.52 | 0.2 | 0.59 | 0.2 |
| Malate | 0.05 | 0.22 | 0.1 | 0.81 | 0 | 0.88 | 0 |
| Citrate | 0.03 | 0.11 | 0.1 | 0.48 | 0.1 | 0.51 | 0.1 |
| Isocitrate | 0.29 | 0.8 | 0 | 0.37 | -0.1 | 0.35 | -0.1 |
| Adenine | 0.32 | 0.22 | 0.2 | 0.44 | 0.1 | 0.49 | 0.1 |
| Hypoxanthine | 0.21 | 0.16 | -0.2 | 0.28 | -0.2 | 0.31 | -0.2 |
| Uric Acid | 0.05 | 0.19 | -0.1 | 0.17 | -0.1 | 0.18 | -0.1 |
| Glycerol | 0.03 | 0.73 | -0.1 | 0.53 | -0.1 | 0.5 | -0.1 |
| Glyceric acid | 8.0e-05 | 0.29 | 0.1 | 0.49 | 0.1 | 0.51 | 0.1 |
| Glycerol-3-phosphate | 0.04 | 0.09 | 0.2 | 0.19 | 0.1 | 0.19 | 0.1 |
| Myo-inositol | 0.42 | 0.13 | -0.1 | 0.07 | -0.1 | 0.07 | -0.1 |
| Glucose | 0.6 | 0.21 | -0.1 | 0.75 | 0 | 0.73 | 0 |
| Quinic acid | 0.12 | 0.74 | 0.1 | 0.85 | 0.1 | 0.77 | 0.1 |
| Xylitol | 0.89 | 0.21 | -0.1 | 0.11 | -0.2 | 0.11 | -0.2 |
| Valine | 0.27 | 0.07 | 0.1 | 0.05 | 0.1 | 0.05 | 0.1 |
| Leucine | 0.09 | 0.16 | 0.1 | 0.13 | 0.1 | 0.13 | 0.1 |
| Isoleucine | 0.92 | 0.52 | 0.1 | 0.37 | 0.1 | 0.35 | 0.1 |
| Alanine | 0.5 | 0.45 | -0.1 | 0.12 | -0.2 | 0.12 | -0.2 |
| Glutamate | 0.05 | 0.34 | -0.2 | 0.83 | 0 | 0.82 | 0 |
| Glutamine | 0.85 | 0.35 | 0.1 | 0.93 | 0 | 0.95 | 0 |
| Glutamine^4^ | 0.28 | 0.72 | 0 | 0.7 | 0 | 0.65 | -0.1 |
| Pyroglutamate | 0.01 | 0.69 | 0 | 0.89 | 0 | 0.89 | 0 |
| Arginine-NH3 | 0.89 | 0.41 | -0.1 | 0.15 | -0.1 | 0.15 | -0.1 |
| Tyrosine | 0.05 | 0.42 | 0.1 | 0.69 | 0 | 0.68 | 0 |
| Phenylalanine | 0.44 | 0.7 | 0 | 0.76 | 0 | 0.79 | 0 |
| Proline | 0.15 | 0.13 | -0.2 | 0.12 | -0.2 | 0.11 | -0.2 |
| Hydroxyproline | 0.15 | 0.22 | -0.2 | 0.36 | -0.1 | 0.34 | -0.1 |
| Tryptophan | 0.57 | 0.16 | 0.1 | 0.19 | 0.1 | 0.17 | 0.1 |
| Indole-3-acetate | 0.26 | 0.21 | 0.2 | 0.22 | 0.2 | 0.16 | 0.2 |
| Taurine | 0.02 | 0.01 | 0.3 | 0.08 | 0.2 | 0.08 | 0.2 |
| Threonine | 0.06 | 0.06 | 0.2 | 0.29 | 0.1 | 0.29 | 0.1 |
| Serine | 0.12 | 0.01 | 0.2 | 0.06 | 0.1 | 0.07 | 0.1 |
| Asparagine | 0.66 | 0.17 | 0.1 | 0.88 | 0 | 0.86 | 0 |
| Lysine | 0.37 | 0.32 | 0.1 | 0.43 | 0 | 0.45 | 0 |
| Cystine^3^ | 2.8e-03 | 0.38 | -0.1 | 0.2 | -0.1 | 0.2 | -0.1 |
| Cystine^4^ | 1.3e-03 | 0.1 | -0.2 | 0.1 | -0.2 | 0.11 | -0.2 |
| Glycine | 0.24 | 0.73 | 0 | 0.84 | 0 | 0.87 | 0 |
| Histidine | 0.92 | 0.14 | 0.1 | 0.59 | 0 | 0.6 | 0 |
| Ornithine3^3^ | 0.7 | 0.33 | -0.1 | 0.11 | -0.1 | 0.12 | -0.1 |
| Ornithine^4^ | 0.99 | 0.57 | -0.1 | 0.28 | -0.1 | 0.26 | -0.1 |
| Creatinine | 0.7 | 0.3 | -0.1 | 0.44 | -0.1 | 0.42 | -0.1 |
| β-Alanine | 0.94 | 0.23 | 0.1 | 0.47 | 0.1 | 0.43 | 0.1 |
| Urea | 0.46 | 0.35 | -0.1 | 0.65 | 0 | 0.69 | 0 |
| C12:0 | 0.3 | 0.9 | 0 | 0.92 | 0 | 0.88 | 0 |
| C14:0 | 0.04 | 0.61 | 0.1 | 0.99 | 0 | 0.96 | 0 |
| C16:0 | 0.13 | 0.39 | -0.2 | 0.33 | -0.2 | 0.3 | -0.2 |
| C17:0 | 0.06 | 0.64 | -0.1 | 0.62 | -0.1 | 0.58 | -0.1 |
| C18:0 | 0.07 | 0.8 | 0 | 0.91 | 0 | 0.87 | 0 |
| C18:1 | 0.03 | 0.67 | -0.1 | 0.55 | -0.1 | 0.51 | -0.1 |
| C18:2 | 0.03 | 0.77 | -0.1 | 0.51 | -0.1 | 0.48 | -0.1 |
| C20:4 | 0.19 | 0.47 | -0.1 | 0.29 | -0.1 | 0.27 | -0.2 |
| Cholesterol | 0.73 | 0.62 | 0 | 0.76 | 0 | 0.73 | 0 |
| α-Tocopherol | 0.46 | 0.58 | 0.1 | 0.84 | 0 | 0.84 | 0 |
| **UHPLC** | p_mw_ | p_A_ | b_A_ | p_B_ | b_B_ | p_C_ | b_C_ |
| Hypoxanthine | 0.14 | 0.08 | -0.2 | 0.16 | -0.2 | 0.15 | -0.2 |
| Pyroglutamate | 0.24 | 0.51 | 0.1 | 0.46 | 0.1 | 0.43 | 0.1 |
| OPHospDLTyrso | 0.93 | 0.85 | 0 | 0.73 | 0 | 0.75 | 0 |
| Phenylalanine | 7.9e-04 | 0.01 | 0.3 | 0.03 | 0.3 | 0.03 | 0.3 |
| Taurine | 0.51 | 0.67 | 0 | 0.64 | 0.1 | 0.63 | 0.1 |
| Lyso-PC 14 | 0.48 | 0.4 | -0.1 | 0.41 | -0.1 | 0.37 | -0.1 |
| Lyso-PC 15 | 0.47 | 0.82 | 0 | 0.66 | 0 | 0.64 | 0 |
| Lyso-PC 16 | 0.09 | 0.21 | -0.1 | 0.44 | 0 | 0.44 | 0 |
| Lyso-PC 16:1 | 0.59 | 0.41 | -0.1 | 0.32 | -0.1 | 0.3 | -0.1 |
| Lyso-PC 17 | 0.72 | 0.95 | 0 | 0.68 | 0 | 0.68 | 0 |
| Lyso-PC 18 | 0.49 | 0.79 | 0 | 0.87 | 0 | 0.84 | 0 |
| Lyso-PC 18:1 | 0.07 | 0.23 | -0.1 | 0.37 | -0.1 | 0.36 | -0.1 |
| PC C32:1 | 0.93 | 0.3 | -0.1 | 0.19 | -0.2 | 0.19 | -0.2 |
| PC C34:1 | 0.84 | 0.47 | -0.1 | 0.24 | -0.1 | 0.24 | -0.1 |
| PC 34:3 | 0.47 | 0.73 | 0 | 0.66 | 0 | 0.67 | 0 |
| PC C36:1 | 0.63 | 0.37 | -0.1 | 0.25 | -0.2 | 0.26 | -0.2 |
| PC C36:2 | 0.17 | 0.13 | -0.2 | 0.13 | -0.2 | 0.13 | -0.2 |
| PC C36:3 | 0.15 | 0.49 | 0 | 0.72 | 0 | 0.75 | 0 |
| PC 36:4 | 0.15 | 0.33 | -0.1 | 0.39 | -0.1 | 0.41 | -0.1 |
| PC C36:5 | 0.85 | 0.84 | 0 | 0.51 | -0.1 | 0.52 | -0.1 |
| PC C38:6 | 0.87 | 0.46 | -0.1 | 0.35 | -0.1 | 0.35 | -0.1 |
| PC C40:6 | 0.97 | 0.96 | 0 | 0.73 | 0 | 0.73 | 0 |
| Carnitine C2 | 0.14 | 0.14 | -0.5 | 0.16 | -0.5 | 0.17 | -0.5 |
| Carnitine C3 | 0.14 | 0.49 | -0.1 | 0.69 | -0.1 | 0.68 | -0.1 |
| Carnitine C4 | 0.06 | 0.09 | -0.3 | 0.13 | -0.3 | 0.12 | -0.3 |
| Carnitine C5 | 0.34 | 0.37 | -0.1 | 0.41 | -0.1 | 0.39 | -0.1 |
| Carnitine C6 | 0.76 | 0.48 | -0.1 | 0.58 | -0.1 | 0.57 | -0.1 |
| Carnitine :8 | 0.91 | 0.48 | -0.2 | 0.7 | -0.1 | 0.68 | -0.1 |
| Carnitine C8:1 | 0.02 | 0.18 | -0.3 | 0.49 | -0.2 | 0.47 | -0.2 |
| Carnitine C10 | 0.93 | 0.56 | -0.1 | 0.67 | -0.1 | 0.65 | -0.1 |
| Carnitine C10:1 | 0.87 | 0.6 | -0.1 | 0.7 | -0.1 | 0.69 | -0.1 |
| Carnitine 10:2 | 0.05 | 0.75 | -0.1 | 0.93 | 0 | 0.94 | 0 |
| Carnitine C12 | 0.81 | 0.5 | -0.1 | 0.51 | -0.1 | 0.51 | -0.1 |
| Carnitine C12:1 | 0.67 | 0.37 | -0.2 | 0.35 | -0.2 | 0.35 | -0.2 |
| Carnitine C14 | 0.51 | 0.96 | 0 | 0.86 | 0 | 0.86 | 0 |
| Carnitine C14:1 | 0.61 | 0.27 | -0.3 | 0.29 | -0.3 | 0.29 | -0.3 |
| Carnitine C14:2 | 0.62 | 0.56 | -0.1 | 0.75 | -0.1 | 0.76 | -0.1 |
| Carnitine C16 | 0.2 | 0.18 | -0.2 | 0.28 | -0.2 | 0.27 | -0.2 |
| Carnitine C18 | 0.16 | 0.16 | -0.2 | 0.39 | -0.1 | 0.37 | -0.1 |
| Carnitine C18:1 | 0.19 | 0.09 | -0.3 | 0.16 | -0.3 | 0.16 | -0.3 |
| Carnitine C18:2 | 0.17 | 0.16 | -0.2 | 0.18 | -0.2 | 0.17 | -0.2 |

pmw: Mann-Whitney test

pA: linear regression A using covariates *source*, *sex, age,*

pB: linear regression B using covariates *source, sex, age, HbA1c,*

pc : linear regression C using covariates *source, sex, age, HbA1c, eGFR (MDRD)*

**Table S2. Associations of untargeted metabolites in the DIALONG study (NP vs RP groups).**

| **GC-MS** | p_mw_ | p_A_ | b_A_ | p_B_ | b_B_ | p_C_ | b_C_ |
| --- | --- | --- | --- | --- | --- | --- | --- |
| α-Hydroxybutyrate | 1 | 0.98 | 0 | 0.73 | -0.1 | 0.59 | -0.1 |
| β-Hydroxybutyrate | 0.91 | 0.95 | 0 | 0.66 | -0.1 | 0.57 | -0.2 |
| Succinate | 0.08 | 0.17 | 0.1 | 0.31 | 0.1 | 0.29 | 0.1 |
| Fumarate | 0.69 | 0.96 | 0 | 0.88 | 0 | 0.86 | 0 |
| Malate | 1 | 0.75 | 0 | 0.91 | 0 | 0.98 | 0 |
| Citrate | 0.42 | 0.49 | 0.2 | 0.48 | 0.2 | 0.36 | 0.2 |
| Isocitrate | 0.45 | 0.69 | 0.1 | 0.62 | 0.1 | 0.46 | 0.2 |
| Uric Acid | 0.05 | 0.05 | -0.2 | 0.11 | -0.1 | 0.13 | -0.1 |
| Glycerol | 0.1 | 0.06 | 0.3 | 0.18 | 0.2 | 0.24 | 0.2 |
| Glyceric acid | 0.39 | 0.6 | 0.1 | 0.58 | 0.1 | 0.53 | 0.1 |
| Glycerol-2-phosphate | 0.06 | 0.05 | -0.5 | 0.13 | -0.4 | 0.15 | -0.4 |
| Glycerol-3-phosphate | 0.33 | 0.28 | -0.4 | 0.35 | -0.3 | 0.43 | -0.3 |
| Myo-inositol | 0.02 | 0.02 | -0.4 | 0.07 | -0.3 | 0.09 | -0.3 |
| Glucose | 0.29 | 0.46 | -0.1 | 0.46 | -0.1 | 0.48 | -0.1 |
| Threonic acid | 0.1 | 0.1 | -0.4 | 0.1 | -0.4 | 0.11 | -0.4 |
| Erythritol | 9.4e-03 | 4.3e-03 | -0.6 | 0.03 | -0.5 | 0.04 | -0.4 |
| Pyrophosphate | 0.05 | 0.07 | -0.4 | 0.15 | -0.3 | 0.18 | -0.3 |
| Valine | 0.43 | 0.48 | -0.1 | 0.38 | -0.1 | 0.32 | -0.1 |
| Leucine | 0.42 | 0.99 | 0 | 0.72 | 0 | 0.61 | -0.1 |
| Isoleucine | 0.17 | 0.23 | -0.1 | 0.27 | -0.1 | 0.22 | -0.1 |
| Alanine | 0.48 | 0.19 | 0.1 | 0.15 | 0.1 | 0.1 | 0.1 |
| Aspartate | 0.12 | 0.07 | -0.3 | 0.11 | -0.3 | 0.11 | -0.3 |
| Glutamate | 8.2e-03 | 0.02 | -0.7 | 0.07 | -0.6 | 0.08 | -0.6 |
| Glutamine^3^ | 0.35 | 0.47 | -0.1 | 0.56 | -0.1 | 0.63 | -0.1 |
| Glutamine^4^ | 0.1 | 0.13 | -0.4 | 0.24 | -0.3 | 0.27 | -0.3 |
| Pyroglutamate | 7.9e-03 | 0.01 | -0.3 | 0.03 | -0.3 | 0.03 | -0.3 |
| Arginine-NH3 | 0.49 | 0.91 | 0 | 0.9 | 0 | 0.8 | 0.1 |
| Tyrosine | 0.3 | 0.21 | -0.2 | 0.28 | -0.2 | 0.29 | -0.2 |
| Phenylalanine | 0.18 | 0.13 | -0.2 | 0.26 | -0.1 | 0.32 | -0.1 |
| Proline | 0.23 | 0.27 | -0.1 | 0.32 | -0.1 | 0.35 | -0.1 |
| Hydroxyproline | 0.02 | 0.02 | -0.5 | 0.05 | -0.4 | 0.07 | -0.4 |
| Tryptophan | 0.21 | 0.2 | -0.2 | 0.21 | -0.3 | 0.21 | -0.3 |
| Taurine | 0.03 | 0.04 | -0.7 | 0.1 | -0.5 | 0.12 | -0.5 |
| Threonine | 0.99 | 0.89 | 0 | 0.78 | 0 | 0.71 | 0 |
| Serine | 0.23 | 0.11 | 0.1 | 0.27 | 0.1 | 0.33 | 0.1 |
| Asparagine | 0.07 | 0.05 | -0.5 | 0.13 | -0.4 | 0.16 | -0.3 |
| Lysine | 0.19 | 0.29 | -0.1 | 0.32 | -0.1 | 0.32 | -0.1 |
| Cystine^3^ | 0.01 | 0.02 | -0.6 | 0.03 | -0.5 | 0.04 | -0.5 |
| Cystine^4^ | 0.06 | 0.03 | -0.8 | 0.05 | -0.8 | 0.06 | -0.8 |
| Glycine | 0.51 | 0.21 | 0.1 | 0.28 | 0.1 | 0.26 | 0.1 |
| Ornithine3^3^ | 0.03 | 0.02 | -0.5 | 0.06 | -0.4 | 0.07 | -0.4 |
| Ornithine^4^ | 0.4 | 0.7 | 0.1 | 0.71 | 0.1 | 0.58 | 0.2 |
| Creatinine | 0.05 | 0.03 | -0.5 | 0.09 | -0.4 | 0.12 | -0.3 |
| Urea | 0.37 | 0.29 | -0.1 | 0.46 | -0.1 | 0.65 | 0 |
| C12:0 | 0.72 | 0.4 | -0.2 | 0.42 | -0.2 | 0.49 | -0.2 |
| C14:0 | 0.3 | 0.23 | 0.4 | 0.44 | 0.2 | 0.38 | 0.3 |
| C16:0 | 0.89 | 0.68 | -0.1 | 0.46 | -0.1 | 0.42 | -0.1 |
| C17:0 | 0.52 | 0.41 | -0.2 | 0.44 | -0.2 | 0.43 | -0.2 |
| C18:0 | 0.48 | 0.49 | -0.1 | 0.35 | -0.2 | 0.37 | -0.2 |
| C18:1 | 0.4 | 0.37 | -0.2 | 0.34 | -0.2 | 0.32 | -0.3 |
| C18:2 | 0.35 | 0.32 | -0.3 | 0.27 | -0.4 | 0.28 | -0.3 |
| C20:4 | 0.31 | 0.37 | -0.3 | 0.24 | -0.4 | 0.28 | -0.4 |
| Cholesterol | 4.8e-03 | 3.1e-03 | 0.3 | 0.01 | 0.3 | 7.9e-03 | 0.3 |
| Octadecanol | 0.05 | 0.08 | -0.3 | 0.12 | -0.3 | 0.12 | -0.3 |
| α-Tocopherol | 0.37 | 0.43 | 0.2 | 0.48 | 0.1 | 0.49 | 0.1 |
| **UHPLC** | p_mw_ | p_A_ | b_A_ | p_B_ | b_B_ | p_C_ | b_C_ |
| Hypoxanthine | 0.86 | 0.8 | 0 | 0.95 | 0 | 0.82 | 0 |
| Caffeine | 0.18 | 0.12 | -0.7 | 0.17 | -0.7 | 0.23 | -0.6 |
| Paraxanthine | 0.15 | 0.15 | -0.5 | 0.18 | -0.5 | 0.25 | -0.4 |
| Uric acid | 0.13 | 0.15 | -0.2 | 0.21 | -0.1 | 0.36 | -0.1 |
| Trans-cinnamic acid | 0.98 | 0.89 | 0 | 0.9 | 0 | 0.78 | 0 |
| Hydroxycinnamic acid | 0.72 | 0.82 | 0 | 0.88 | 0 | 0.93 | 0 |
| Hippuric acid | 0.02 | 0.02 | -0.6 | 0.07 | -0.5 | 0.12 | -0.4 |
| L-valine | 0.37 | 0.18 | -0.1 | 0.43 | -0.1 | 0.3 | -0.1 |
| Leucine-Isoleucine | 0.36 | 0.5 | -0.1 | 0.45 | -0.1 | 0.39 | -0.1 |
| Pyroglutamate | 0.19 | 0.4 | -0.1 | 0.38 | -0.1 | 0.44 | -0.1 |
| L-Tyrosine | 0.74 | 0.81 | 0 | 0.87 | 0 | 0.92 | 0 |
| L-Phenylalanine | 0.23 | 0.34 | 0.1 | 0.38 | 0.1 | 0.38 | 0.1 |
| Phenylacetamide | 0.7 | 0.81 | 0 | 0.88 | 0 | 0.92 | 0 |
| L-Proline | 0.38 | 0.45 | -0.1 | 0.51 | -0.1 | 0.66 | 0 |
| L-Tryptophan | 0.17 | 0.3 | 0.1 | 0.33 | 0.1 | 0.33 | 0.1 |
| Indole | 0.49 | 0.46 | -0.3 | 0.63 | -0.2 | 0.51 | -0.3 |
| L-Methionine | 0.83 | 0.98 | 0 | 0.93 | 0 | 0.8 | 0 |
| Carnitine | 0.06 | 0.03 | -0.1 | 0.06 | -0.1 | 0.08 | -0.1 |
| LPC 16:0 | 0.54 | 0.56 | -0.1 | 0.35 | -0.2 | 0.33 | -0.2 |
| LPC 18:0 | 0.23 | 0.18 | -0.2 | 0.15 | -0.3 | 0.13 | -0.3 |
| LPC 18:2 | 0.58 | 0.66 | -0.1 | 0.65 | -0.1 | 0.67 | -0.1 |
| LPC 20:0 | 0.9 | 0.93 | 0 | 0.59 | 0.2 | 0.61 | 0.2 |
| LPC 20:3 | 0.7 | 0.62 | -0.1 | 0.4 | -0.1 | 0.41 | -0.1 |
| LPC 20:4 | 0.28 | 0.27 | -0.1 | 0.28 | -0.1 | 0.25 | -0.1 |
| LPC 20:5 | 0.39 | 0.43 | -0.1 | 0.42 | -0.1 | 0.41 | -0.1 |
| LPC 22:5 | 0.25 | 0.26 | -0.2 | 0.23 | -0.2 | 0.23 | -0.2 |
| LPC 22:6 | 0.13 | 0.1 | -0.3 | 0.13 | -0.3 | 0.12 | -0.3 |
| LPE C20:4 | 0.02 | 9.7e-03 | -0.7 | 0.03 | -0.6 | 0.03 | -0.6 |
| LPE C20:5 | 0.14 | 0.11 | -0.2 | 0.14 | -0.2 | 0.13 | -0.2 |
| LPE C22:6 | 0.23 | 0.1 | -0.2 | 0.14 | -0.2 | 0.15 | -0.2 |
| PC 34:1 | 0.3 | 0.36 | -0.1 | 0.27 | -0.1 | 0.24 | -0.1 |
| PC 36:4 | 0.63 | 0.63 | 0 | 0.56 | 0 | 0.42 | 0 |
| PC 38:4 | 0.61 | 0.59 | -0.1 | 0.4 | -0.1 | 0.39 | -0.1 |
| PC 38:5 | 0.98 | 0.75 | 0 | 0.51 | 0 | 0.63 | 0 |
| SM 36:1 | 0.45 | 0.76 | -0.1 | 0.63 | -0.1 | 0.61 | -0.1 |
| Carnitine C2 | 0.56 | 0.46 | -0.1 | 0.31 | -0.1 | 0.31 | -0.1 |
| Carnitine C3 | 0.48 | 0.38 | -0.1 | 0.4 | -0.1 | 0.53 | -0.1 |
| Carnitine C4 | 2.2e-03 | 2.4e-03 | -0.5 | 9.1e-03 | -0.4 | 0.01 | -0.4 |
| Carnitine C5 | 0.03 | 6.1e-03 | -0.6 | 0.01 | -0.6 | 0.01 | -0.6 |
| Carnitine C6 | 0.78 | 0.64 | -0.1 | 0.67 | -0.1 | 0.71 | -0.1 |
| Carnitine C8 | 0.86 | 0.88 | 0 | 0.99 | 0 | 0.91 | 0 |
| Carnitine C8:1 | 0.76 | 0.82 | 0 | 0.63 | 0.1 | 0.29 | 0.1 |
| Carnitine C10 | 0.43 | 0.46 | 0.1 | 0.44 | 0.2 | 0.41 | 0.2 |
| Carnitine C10:1 | 0.75 | 0.77 | 0.1 | 0.62 | 0.1 | 0.54 | 0.1 |
| Carnitine C10:2 | 0.75 | 0.55 | 0.1 | 0.38 | 0.1 | 0.28 | 0.1 |
| Carnitine C12 | 0.27 | 0.34 | 0.2 | 0.33 | 0.2 | 0.26 | 0.2 |
| Carnitine C12:1 | 0.56 | 0.58 | 0.1 | 0.47 | 0.1 | 0.34 | 0.2 |
| Carnitine C14 | 0.64 | 0.71 | 0.1 | 0.8 | 0 | 0.71 | 0.1 |
| Carnitine C14:1 | 0.85 | 0.82 | 0 | 0.76 | 0.1 | 0.67 | 0.1 |
| Carnitine C14:2 | 0.68 | 0.72 | 0.1 | 0.61 | 0.1 | 0.46 | 0.1 |

pmw: Mann-Whitney test

pA: linear regression A using covariates *sex, age,*

pB: linear regression B using covariates *sex, age, HbA1c,*

pc : linear regression C using covariates *sex, age, HbA1c, eGFR (MDRD)*

**Table S3. Associations of targeted metabolites in the DIALONG study (NP vs RP groups).**

|  | NP  Mean (sd) | RP  Mean (sd) | p_mw_ | p_A_ | b_A_ | p_B_ | b_B_ | p_C_ | b_C_ |  |
| --- | --- | --- | --- | --- | --- | --- | --- | --- | --- | --- |
| Tot. homocysteine | 10.5 (2.2) | 11.2 (3.9) | 1.00 | 0.28 | -0.1 | 0.40 | -0.1 | 0.72 | 0 |  |
| Tot. cysteine | 301.6 (35.8) | 311.4 (47.9) | 0.49 | 0.22 | -0.1 | 0.34 | -0.1 | 0.56 | 0 |  |
| Methylmalonic acid | 1.2 (5.7e-02) | 1.2 (5.5e-02) | 0.47 | 0.46 | 0 | 0.54 | 0 | 0.84 | 0 |  |
| Cystathionine | 1.3 (0.2) | 1.3 (0.2) | 0.25 | 0.28 | 0 | 0.26 | -0.1 | 0.40 | 0 |  |
| Serine | 128.0 (26.7) | 113.7 (27.7) | 0.09 | 0.02 | 0.2 | 0.03 | 0.2 | 0.03 | 0.2 |  |
| Glycine | 314.1 (58.3) | 275.6 (45.8) | 0.02 | 0.01 | 0.2 | 0.01 | 0.2 | 4e-03 | 0.2 |  |
| Histidine | 75.0 (7.5) | 72.2 (6.3) | 0.14 | 0.12 | 0.1 | 0.20 | 0 | 0.17 | 0.1 |  |
| Ornithine | 66.5 (17.4) | 66.9 (18.6) | 0.99 | 0.84 | 0 | 0.68 | 0 | 0.79 | 0 |  |
| Aspartate | 6.9 (1.9) | 7.4 (2.3) | 0.74 | 0.40 | -0.1 | 0.46 | -0.1 | 0.35 | -0.1 |  |
| Glutamate | 66.0 (27.6) | 78.8 (33.7) | 0.17 | 0.15 | -0.2 | 0.19 | -0.2 | 0.17 | -0.2 |  |
| Lysine | 177.3 (28.6) | 173.2 (25.3) | 0.65 | 0.66 | 0 | 0.80 | 0 | 0.70 | 0 |  |
| Alanine | 326.2 (65.6) | 306.6 (60.7) | 0.42 | 0.33 | 0.1 | 0.30 | 0.1 | 0.13 | 0.1 |  |
| Phenylalanine | 63.6 (7.2) | 59.0 (7.6) | 0.01 | 0.03 | 0.1 | 0.04 | 0.1 | 0.02 | 0.1 |  |
| Isoleucine | 67.2 (14.3) | 67.6 (15.0) | 0.96 | 0.77 | 0 | 0.71 | 0 | 0.68 | 0 |  |
| Leucine | 126.3 (23.5) | 123.7 (23.3) | 0.64 | 0.90 | 0 | 0.85 | 0 | 0.76 | 0 |  |
| Proline | 164.3 (48.0) | 163.3 (39.2) | 0.94 | 0.75 | 0 | 0.69 | 0 | 0.78 | 0 |  |
| Valine | 262.2 (46.6) | 252.0 (39.1) | 0.41 | 0.60 | 0 | 0.86 | 0 | 0.84 | 0 |  |
| Asparagine | 58.4 (10.4) | 52.8 (8.8) | 0.05 | 0.05 | 0.1 | 0.07 | 0.1 | 0.06 | 0.1 |  |
| Glutamine | 570.8 (80.8) | 575.9 (81.9) | 0.94 | 0.69 | 0 | 0.50 | 0 | 0.46 | 0 |  |
| Threonine | 135.7 (30.0) | 134.6 (37.2) | 0.68 | 0.79 | 0 | 0.92 | 0 | 0.95 | 0 |  |
| Tyrosine | 67.7 (8.7) | 65.1 (9.8) | 0.16 | 0.38 | 0.1 | 0.45 | 0 | 0.44 | 0 |  |
| Tryptophan | 67.2 (11.5) | 62.5 (14.2) | 0.12 | 0.16 | 0.1 | 0.22 | 0.1 | 0.25 | 0.1 |  |
| α-ketoglutaric acid | 9.8 (1.9) | 10.8 (2.4) | 0.23 | 0.13 | -0.1 | 0.16 | -0.1 | 0.16 | -0.1 |  |
| 3-hydroxyisobutyrate | 22.0 (6.0) | 20.8 (6.2) | 0.28 | 0.45 | 0.1 | 0.60 | 0.1 | 0.54 | 0.1 |  |
| α-hydroxybutyrate | 42.0 (18.4) | 47.3 (21.9) | 0.25 | 0.34 | -0.2 | 0.37 | -0.2 | 0.19 | -0.2 |  |
| β-hydroxybutyrate | 202.3 (255.0) | 222.2 (196.2) | 0.56 | 0.77 | -0.1 | 0.68 | -0.2 | 0.54 | -0.2 |  |
| Methionine | 30.2 (5.6) | 27.3 (4.5) | 0.06 | 0.05 | 0.1 | 0.08 | 0.1 | 0.05 | 0.1 |  |
| Kynurenine | 2.5 (0.3) | 2.5 (0.4) | 0.73 | 0.80 | 0 | 0.93 | 0 | 0.61 | 0 |  |
| Tryptophan | 65.1 (12.0) | 59.9 (13.7) | 0.09 | 0.12 | 0.1 | 0.19 | 0.1 | 0.21 | 0.1 | |
| Kynurenine | 2.7 (0.4) | 2.6 (0.5) | 0.49 | 0.83 | 0.0 | 0.70 | 0.0 | 0.29 | 0.1 | |
| Kynurenic acid | 52.3 (17.3) | 57.1 (22.7) | 0.67 | 0.46 | -0.1 | 0.48 | -0.1 | 0.90 | 0.0 | |
| Anthranilic acid | 17.1 (4.3) | 18.2 (6.7) | 0.90 | 0.52 | -0.1 | 0.49 | -0.1 | 0.63 | -0.1 | |
| 3-hydroxykynurenine | 46.0 (13.4) | 51.4 (22.9) | 0.74 | 0.41 | -0.1 | 0.52 | -0.1 | 0.84 | 0.0 | |
| Xanthurenic acid | 14.6 (4.5) | 17.2 (6.6) | 0.35 | 0.10 | -0.2 | 0.08 | -0.3 | 0.13 | -0.2 | |
| 3-hydroxyanthranilic acid | 31.0 (13.2) | 29.8 (8.1) | 0.80 | 0.69 | -0.1 | 0.53 | -0.1 | 0.71 | 0.0 | |
| Picolinic acid | 57.0 (19.7) | 49.7 (21.3) | 0.07 | 0.13 | 0.2 | 0.18 | 0.2 | 0.10 | 0.2 | |
| Quinolinc acid | 350.3 (124.8) | 398.9 (179.6) | 0.77 | 0.22 | -0.2 | 0.33 | -0.1 | 0.56 | -0.1 | |
| Neopterin | 10.8 (2.9) | 11.8 (4.6) | 1.00 | 0.42 | -0.1 | 0.54 | -0.1 | 0.94 | 0.0 | |
| Nicotinamide | 207.7 (50.0) | 214.4 (71.0) | 0.79 | 0.86 | 0.0 | 0.56 | -0.1 | 0.50 | -0.1 | |
| N1-methylnicotinamide | 108.5 (63.5) | 129.9 (77.4) | 0.27 | 0.31 | -0.2 | 0.19 | -0.3 | 0.25 | -0.2 | |
| Thiamine | 4.4 (3.1) | 5.3 (2.9) | 0.04 | 0.12 | -0.3 | 0.15 | -0.3 | 0.16 | -0.3 | |
| Thiamine monophosphate | 7.9 (3.1) | 10.0 (3.4) | 0.02 | 0.02 | -0.3 | 0.02 | -0.3 | 0.03 | -0.3 | |
| Total thiamine* | 11.5 (6.4) | 14.8 (7.0) | 0.03 | 0.03 | -0.4 | 0.04 | -0.4 | 0.05 | -0.4 | |
| Riboflavin | 13.3 (7.0) | 14.7 (9.2) | 0.72 | 0.85 | 0.0 | 0.89 | 0.0 | 0.89 | 0.0 | |
| Flavin mononucleotide | 12.9 (4.6) | 12.9 (4.1) | 0.81 | 0.98 | 0.0 | 0.88 | 0.0 | 0.93 | 0.0 | |
| Pyridoxal 5’-phosphate | 64.3 (54.6) | 69.2 (61.5) | 0.96 | 0.89 | 0.0 | 0.78 | -0.1 | 0.79 | -0.1 | |
| Pyridoxal | 13.3 (7.7) | 14.5 (10.0) | 0.85 | 0.85 | 0.0 | 0.81 | -0.1 | 0.85 | 0.0 | |
| 4-pyridoxic acid | 28.3 (16.6) | 35.4 (21.6) | 0.40 | 0.23 | -0.3 | 0.25 | -0.3 | 0.35 | -0.2 | |
| Trigonelline | 3.1 (2.0) | 4.5 (3.3) | 0.08 | 0.08 | -0.4 | 0.11 | -0.4 | 0.18 | -0.3 | |
| Cystathionine | 1.3 (0.2) | 1.4 (0.2) | 0.34 | 0.30 | -0.1 | 0.28 | -0.1 | 0.43 | 0.0 | |

pmw: Mann-Whitney test

pA: linear regression A using covariates *sex, age,*

pB: linear regression B using covariates *sex, age, HbA1c,*

pc : linear regression C using covariates *sex, age, HbA1c, eGFR (MDRD)*

**Table S4.** DIALONG phenotype associations (T1D vs Control)

|  | n_control_ | N_T1D_ | Control | T1D | p_mw_ | p_A_ | p_B_ | p_C_ |
| --- | --- | --- | --- | --- | --- | --- | --- | --- |
| *Treatment* | | | | | | | | |
| Statin | 72 | 71 | 10 (14%) | 34 (48%) | 2e-05 |  |  |  |
| β-blocker | 72 | 71 | 1 (1%) | 13 (18%) | 2e-03 |  |  |  |
| Anti platelet | 72 | 71 | 8 (11%) | 19 (27%) | 0.03 |  |  |  |
| Diuretic loop | 72 | 71 | 4 (6%) | 8 (11%) | 0.35 |  |  |  |
| ACE/ARB | 72 | 71 | 14 (19%) | 31 (44%) | 3e-03 |  |  |  |
| *Clinical characteristics* | | | | | | | | |
| Sex | 72 | 71 | 32 (44%) | 29 (41%) | 0.79 |  |  |  |
| Smoking | 72 | 71 | 6 (8%) | 3 (4%) | 0.50 |  |  |  |
| Hypertension | 72 | 71 | 34 (47%) | 42 (59%) | 0.21 |  |  |  |
| GAD pos. | 70 | 68 | 0 (0%) | 0 (0%) |  |  |  |  |
| Hypercholesterolaemia | 72 | 71 | 15 (21%) | 23 (32%) | 0.17 |  |  |  |
| Age, visit [years] | 72 | 71 | 62.6 (6.9) | 61.3 (6.9) | 0.25 |  |  |  |
| HbA1c (%) | 68 | 70 | 5.5 (0.3) | 7.4 (0.8) | 6.2e-24 | 1.6e-42 |  |  |
| eGDR | 68 | 70 | 8.3 (2.3) | 6.9 (2.0) | 6.7e-04 | 2.8e-05 | 0.19 | 0.33 |
| C-peptide | 70 | 68 | 712.8 (195.1) | 8.7 (38.5) | 6.7e-26 | 6.9e-60 | 3.6e-29 | 1.3e-28 |
| SysBP | 72 | 71 | 136.8 (19.1) | 144.7 (18.4) | 8.3e-03 | 4.9e-03 | 1.1e-03 | 3.9e-03 |
| DiasBP | 72 | 71 | 81.3 (9.2) | 74.5 (7.9) | 1.4e-05 | 9.3e-06 | 0.73 | 0.53 |
| Height | 72 | 71 | 172.4 (9.6) | 171.1 (10.1) | 0.53 | 0.39 | 0.04 | 0.03 |
| Weight | 72 | 71 | 76.8 (14.8) | 75.8 (14.1) | 0.84 | 0.81 | 0.34 | 0.39 |
| Waist | 72 | 71 | 89.8 (12.7) | 89.6 (12.5) | 0.99 | 0.83 | 0.05 | 0.05 |
| BMI | 72 | 71 | 25.8 (4.3) | 25.8 (3.7) | 0.72 | 0.93 | 0.02 | 0.03 |
| Cholesterol | 70 | 70 | 5.8 (1.2) | 5.0 (1.0) | 5.3e-05 | 1.1e-05 | 0.50 | 0.71 |
| HDL | 70 | 70 | 1.7 (0.5) | 2.1 (0.5) | 2.2e-05 | 1.3e-05 | 9.7e-04 | 1.6e-03 |
| LDL | 70 | 70 | 3.8 (1.0) | 2.7 (0.8) | 3.1e-09 | 4.2e-10 | 0.04 | 0.08 |
| Triglycerides | 70 | 70 | 1.1 (0.6) | 0.8 (0.4) | 3.8e-04 | 2.5e-03 | 0.05 | 0.07 |
| TRL | 70 | 70 | 0.3 (0.2) | 0.2 (0.2) | 3.8e-05 | 2.2e-04 | 0.05 | 0.09 |
| GAD | 70 | 68 | 4.5e-02 (0.2) | 0.2 (0.5) | 1.4e-04 | 0.04 | 0.07 | 0.08 |
| Anti-IA2 | 70 | 67 | 7.1e-04 (6.0e-03) | 3.1e-02 (9.0e-02) | 1.9e-03 | 7.0e-03 | 0.13 | 0.15 |
| Anti-ZnT8 | 70 | 68 | 0.0e+00 (0.0e+00) | 2.9e-03 (1.4e-02) | 0.04 | 0.09 | 0.64 | 0.52 |
| Anti-Insulin | 70 | 68 | 8.6e-04 (7.2e-03) | 0.5 (0.8) | 1.3e-17 | 9.3e-08 | 0.04 | 0.02 |
| CRP | 69 | 69 | 1.7 (2.0) | 2.5 (2.6) | 0.08 | 0.04 | 0.39 | 0.65 |
| eGFR (MDRD) | 71 | 70 | 80.4 (14.4) | 83.9 (20.8) | 0.17 | 0.25 | 0.02 |  |
| ASAT | 69 | 70 | 24.9 (7.3) | 28.3 (9.7) | 0.07 | 0.02 | 0.35 | 0.35 |
| ALAT | 70 | 70 | 25.2 (11.0) | 27.3 (13.5) | 0.50 | 0.31 | 0.82 | 0.73 |
| GGT | 70 | 70 | 29.9 (20.2) | 31.0 (23.7) | 0.80 | 0.63 | 0.07 | 0.07 |
| ALP | 70 | 70 | 63.7 (18.0) | 75.5 (25.5) | 0.01 | 1.3e-03 | 0.78 | 0.82 |
| ASAT/ALAT | 69 | 70 | 1.1 (0.3) | 1.1 (0.3) | 0.51 | 0.37 | 0.08 | 0.06 |
| HSI | 69 | 70 | 35.1 (6.0) | 34.7 (4.5) | 0.94 | 0.64 | 0.02 | 0.02 |
| FLI | 70 | 70 | 1.2 (1.3) | 1.0 (1.3) | 0.28 | 0.40 | 8.5e-03 | 8.7e-03 |

**Table S5. Associations of untargeted metabolites in the DIALONG study (T1D vs Control groups).**

| **GC-MS** | p_mw_ | p_A_ | b_A_ | p_B_ | b_B_ | p_C_ | b_C_ |
| --- | --- | --- | --- | --- | --- | --- | --- |
| α-Hydroxybutyrate | 0.04 | 0.2 | 0.04 | 0.4 | 0.09 | 0.2 | 0.28 |
| β-Hydroxybutyrate | 6.4e-09 | 1.3 | 5.7e-10 | 1.9 | 1.8e-06 | 1.8 | 1.1e-05 |
| Succinate | 0.86 | 0 | 0.97 | 0.1 | 0.29 | 0.1 | 0.31 |
| Fumarate | 0.3 | -0.1 | 0.38 | 0 | 0.82 | 0 | 0.96 |
| Malate | 0.55 | 0 | 0.69 | -0.1 | 0.73 | -0.1 | 0.7 |
| Citrate | 0.2 | 0.2 | 0.19 | 0.2 | 0.52 | 0.4 | 0.29 |
| Isocitrate | 0.31 | 0.2 | 0.19 | 0.1 | 0.7 | 0.3 | 0.35 |
| Uric Acid | 0.1 | 0.1 | 0.03 | 0 | 0.71 | 0 | 0.85 |
| Glycerol | 0.03 | 0.2 | 0.03 | 0.5 | 3.6e-03 | 0.4 | 0.02 |
| Glyceric acid | 0.27 | 0.1 | 0.18 | 0.2 | 0.43 | 0.2 | 0.42 |
| Glycerol-2-phosphate | 0.19 | 0.2 | 0.24 | -0.4 | 0.2 | -0.4 | 0.27 |
| Glycerol-3-phosphate | 0.25 | 0.3 | 0.18 | 0 | 0.94 | 0.1 | 0.78 |
| Myo-inositol | 2.1e-06 | 0.5 | 1.3e-05 | 0 | 0.95 | 0.1 | 0.81 |
| Glucose | 3.0e-09 | 0.5 | 5.8e-09 | 0.4 | 8.7e-03 | 0.4 | 0.01 |
| Threonic acid | 1 | 0 | 0.9 | 0.2 | 0.62 | 0.1 | 0.7 |
| Erythritol | 0.94 | 0 | 0.93 | -0.7 | 0.01 | -0.6 | 0.03 |
| Pyrophosphate | 0.24 | -0.2 | 0.11 | -0.6 | 0.05 | -0.5 | 0.09 |
| Valine | 0.23 | 0 | 0.6 | 0 | 1 | -0.1 | 0.66 |
| Leucine | 0.55 | 0 | 0.72 | 0.1 | 0.38 | 0.1 | 0.67 |
| Isoleucine | 0.55 | 0.1 | 0.22 | 0 | 0.86 | 0 | 0.76 |
| Alanine | 4.8e-03 | -0.2 | 5.7e-03 | -0.3 | 0.03 | -0.2 | 0.08 |
| Aspartate | 0.24 | 0.1 | 0.29 | -0.1 | 0.58 | -0.2 | 0.45 |
| Glutamate | 0.79 | 0.1 | 0.72 | -0.7 | 0.11 | -0.7 | 0.1 |
| Glutamine^3^ | 0.67 | -0.1 | 0.61 | -0.2 | 0.53 | -0.1 | 0.77 |
| Glutamine^4^ | 0.81 | 0.1 | 0.72 | -0.4 | 0.28 | -0.3 | 0.39 |
| Pyroglutamate | 0.97 | 0 | 0.78 | -0.2 | 0.3 | -0.2 | 0.25 |
| Arginine-NH3 | 0.18 | -0.1 | 0.37 | -0.1 | 0.81 | 0 | 0.86 |
| Tyrosine | 1.1e-03 | 0.3 | 4.1e-03 | 0.1 | 0.61 | 0.1 | 0.76 |
| Phenylalanine | 0.99 | 0 | 0.73 | -0.2 | 0.35 | -0.2 | 0.42 |
| Proline | 0.59 | 0 | 0.71 | -0.2 | 0.29 | -0.2 | 0.36 |
| Hydroxyproline | 0.05 | 0.3 | 0.05 | -0.1 | 0.69 | -0.1 | 0.81 |
| Tryptophan | 3.6e-07 | 0.9 | 9.9e-09 | 0.8 | 8.7e-03 | 0.8 | 0.01 |
| Taurine | 0.18 | -0.3 | 0.21 | -1.1 | 0.01 | -1 | 0.03 |
| Threonine | 0.61 | -0.1 | 0.48 | 0.1 | 0.72 | 0 | 0.98 |
| Serine | 0.53 | 0 | 0.88 | 0.2 | 0.07 | 0.1 | 0.17 |
| Asparagine | 0.88 | 0 | 0.9 | -0.5 | 0.11 | -0.5 | 0.15 |
| Lysine | 0.18 | 0.1 | 0.28 | 0 | 0.79 | 0 | 0.87 |
| Cystine^3^ | 0.2 | -0.2 | 0.39 | -0.5 | 0.19 | -0.5 | 0.19 |
| Cystine^4^ | 0.02 | 0.8 | 5.4e-03 | 0.2 | 0.65 | 0.3 | 0.62 |
| Glycine | 0.4 | 0 | 0.9 | 0.1 | 0.42 | 0.1 | 0.25 |
| Ornithine3^3^ | 0.27 | 0.1 | 0.5 | -0.3 | 0.39 | -0.3 | 0.42 |
| Ornithine^4^ | 0.45 | 0 | 0.78 | 0 | 0.96 | 0.2 | 0.61 |
| Creatinine | 0.68 | 0.1 | 0.75 | -0.5 | 0.12 | -0.4 | 0.21 |
| Urea | 0.88 | 0 | 0.62 | -0.1 | 0.22 | 0 | 0.65 |
| C12:0 | 0.15 | 0.3 | 0.12 | 0.2 | 0.64 | 0.2 | 0.56 |
| C14:0 | 0.5 | -0.1 | 0.65 | 0.4 | 0.38 | 0.4 | 0.3 |
| C16:0 | 3.6e-04 | 0.3 | 3.7e-04 | 0.4 | 0.01 | 0.4 | 0.03 |
| C17:0 | 9.5e-08 | 1.1 | 4.5e-09 | 0.9 | 0.01 | 0.8 | 0.03 |
| C18:0 | 3.4e-07 | 0.7 | 9.7e-08 | 0.8 | 2.0e-03 | 0.8 | 2.9e-03 |
| C18:1 | 4.5e-06 | 0.8 | 4.5e-06 | 0.9 | 0.01 | 0.8 | 0.02 |
| C18:2 | 3.9e-07 | 1.1 | 6.3e-08 | 1.3 | 1.8e-03 | 1.3 | 2.4e-03 |
| C20:4 | 2.4e-07 | 1.1 | 7.5e-08 | 1.4 | 7.5e-04 | 1.4 | 4.5e-04 |
| Cholesterol | 0.05 | -0.2 | 0.05 | 0.1 | 0.42 | 0.2 | 0.22 |
| Octadecanol | 1.1e-07 | 0.8 | 6.7e-09 | 0.5 | 0.07 | 0.5 | 0.09 |
| α-Tocopherol | 0.83 | 0 | 0.75 | 0.1 | 0.66 | 0.1 | 0.66 |
| **UHPLC** | p_mw_ | p_A_ | b_A_ | p_B_ | b_B_ | p_C_ | b_C_ |
| Hypoxanthine | 0.32 | 0.1 | 0.19 | 0.1 | 0.44 | 0.2 | 0.4 |
| Caffeine | 0.14 | -0.4 | 0.17 | -0.5 | 0.44 | -0.2 | 0.78 |
| Paraxanthine | 0.3 | -0.3 | 0.18 | -0.2 | 0.57 | 0 | 0.97 |
| Uric acid | 0.1 | -0.1 | 0.22 | -0.2 | 0.12 | -0.1 | 0.42 |
| Trans-cinnamic acid | 0.22 | 0.1 | 0.15 | 0 | 0.65 | 0 | 0.59 |
| Hydroxycinnamic acid | 0.62 | 0 | 0.39 | 0 | 0.86 | 0 | 0.76 |
| Hippuric acid | 0.56 | 0.1 | 0.74 | -0.5 | 0.2 | -0.1 | 0.7 |
| L-valine | 1.9e-03 | 0.2 | 8.4e-04 | -0.1 | 0.34 | -0.1 | 0.17 |
| Leucine-Isoleucine | 0.23 | 0.1 | 0.11 | 0.1 | 0.39 | 0.1 | 0.6 |
| Pyroglutamate | 0.2 | 0.1 | 0.22 | 0.1 | 0.66 | 0.1 | 0.58 |
| L-Tyrosine | 0.35 | 0.1 | 0.28 | 0 | 0.93 | 0 | 0.83 |
| L-Phenylalanine | 0.92 | 0 | 0.93 | 0 | 0.93 | 0 | 0.89 |
| Phenylacetamide | 0.56 | 0 | 0.4 | 0 | 0.82 | 0 | 0.71 |
| L-Proline | 0.54 | 0 | 0.55 | -0.1 | 0.28 | -0.1 | 0.56 |
| L-Tryptophan | 0.79 | 0 | 0.98 | 0 | 0.92 | 0 | 0.9 |
| Indole | 0.9 | 0 | 0.92 | -0.2 | 0.68 | -0.3 | 0.6 |
| L-Methionine | 0.12 | 0.1 | 0.12 | 0 | 0.71 | 0.1 | 0.56 |
| Carnitine | 1.2e-04 | -0.2 | 5.6e-05 | -0.3 | 8.6e-04 | -0.3 | 1.7e-03 |
| LPC 16:0 | 0.12 | 0.2 | 0.03 | 0.5 | 0.02 | 0.5 | 0.03 |
| LPC 18:0 | 0.31 | 0.2 | 0.15 | 0.3 | 0.23 | 0.3 | 0.32 |
| LPC 18:2 | 0.03 | 0.3 | 5.4e-03 | 0.3 | 0.1 | 0.3 | 0.11 |
| LPC 20:0 | 0.7 | -0.1 | 0.55 | -0.6 | 0.11 | -0.6 | 0.13 |
| LPC 20:3 | 0.96 | 0 | 0.61 | 0.2 | 0.24 | 0.2 | 0.22 |
| LPC 20:4 | 0.02 | 0.2 | 0.01 | 0.1 | 0.52 | 0.1 | 0.6 |
| LPC 20:5 | 0.07 | 0.1 | 0.04 | 0.1 | 0.46 | 0.1 | 0.45 |
| LPC 22:5 | 0.19 | 0.1 | 0.15 | 0.1 | 0.51 | 0.1 | 0.5 |
| LPC 22:6 | 0.58 | 0 | 0.72 | -0.2 | 0.53 | -0.2 | 0.47 |
| LPE C20:4 | 8.8e-03 | 0.5 | 0.01 | -0.3 | 0.38 | -0.3 | 0.38 |
| LPE C20:5 | 0.18 | 0.1 | 0.25 | 0 | 0.93 | 0 | 0.91 |
| LPE C22:6 | 0.58 | 0.1 | 0.29 | 0 | 0.88 | 0 | 0.92 |
| PC 34:1 | 0.69 | 0 | 0.67 | 0 | 0.92 | 0 | 0.82 |
| PC 36:4 | 0.34 | 0 | 0.28 | 0 | 0.56 | -0.1 | 0.3 |
| PC 38:4 | 0.96 | 0 | 0.93 | 0.1 | 0.68 | 0 | 0.82 |
| PC 38:5 | 0.16 | 0.1 | 0.16 | 0.1 | 0.07 | 0.2 | 0.03 |
| SM 36:1 | 0.36 | -0.1 | 0.39 | 0 | 0.95 | -0.1 | 0.78 |
| Carnitine C2 | 0.13 | 0.1 | 0.08 | 0.3 | 0.09 | 0.3 | 0.09 |
| Carnitine C3 | 1.3e-03 | -0.2 | 7.6e-04 | -0.4 | 9.8e-03 | -0.3 | 0.02 |
| Carnitine C4 | 0.1 | 0.2 | 0.02 | -0.3 | 0.14 | -0.3 | 0.13 |
| Carnitine C5 | 0.2 | 0.3 | 0.03 | -0.2 | 0.41 | -0.2 | 0.41 |
| Carnitine C6 | 0.17 | 0.2 | 0.04 | 0.2 | 0.37 | 0.2 | 0.4 |
| Carnitine C8 | 0.32 | 0.2 | 0.14 | 0.1 | 0.8 | 0.1 | 0.68 |
| Carnitine C8:1 | 0.44 | 0.1 | 0.45 | -0.1 | 0.62 | 0 | 0.77 |
| Carnitine C10 | 0.18 | 0.2 | 0.1 | 0.2 | 0.41 | 0.2 | 0.4 |
| Carnitine C10:1 | 0.08 | 0.3 | 0.03 | 0.2 | 0.46 | 0.2 | 0.35 |
| Carnitine C10:2 | 0.52 | 0.1 | 0.42 | 0 | 0.9 | 0 | 0.81 |
| Carnitine C12 | 0.82 | 0.1 | 0.66 | 0 | 0.89 | 0.1 | 0.81 |
| Carnitine C12:1 | 0.23 | 0.2 | 0.14 | 0.1 | 0.75 | 0.1 | 0.55 |
| Carnitine C14 | 0.48 | 0.1 | 0.3 | 0.2 | 0.37 | 0.2 | 0.37 |
| Carnitine C14:1 | 0.16 | 0.2 | 0.07 | 0.2 | 0.38 | 0.2 | 0.33 |
| Carnitine C14:2 | 0.12 | 0.2 | 0.06 | 0.1 | 0.5 | 0.2 | 0.32 |

**Table S6. Associations of targeted metabolites in the DIALONG study (T1D vs Control groups).**

|  | Control  Mean (sd) | T1D  Mean (sd) | p_mw_ | p_A_ | b_A_ | p_B_ | b_B_ | p_C_ | b_C_ |
| --- | --- | --- | --- | --- | --- | --- | --- | --- | --- |
| Tot. homocysteine | 11.2 (2.1) | 10.9 (3.2) | 0.16 | 0.38 | -0.1 | 0.03 | -0.3 | 0.13 | -0.2 |
| Tot. cysteine | 301.6 (27.9) | 306.7 (42.4) | 0.79 | 0.59 | 0.0 | 0.06 | -0.1 | 0.17 | -0.1 |
| Methylmalonic acid | 1.2 (4.0e-02) | 1.2 (5.5e-02) | 0.39 | 0.80 | 0.0 | 0.31 | 0.0 | 0.73 | 0.0 |
| Cystathionine | 1.3 (0.1) | 1.3 (0.2) | 0.73 | 0.72 | 0.0 | 0.65 | 0.0 | 0.29 | 0.1 |
| Serine | 114.0 (16.9) | 120.6 (27.9) | 0.18 | 0.46 | 0.1 | 0.02 | 0.3 | 0.02 | 0.3 |
| Glycine | 287.5 (80.7) | 294.1 (55.2) | 0.37 | 0.32 | 0.1 | 0.19 | 0.2 | 0.09 | 0.2 |
| Histidine | 76.6 (7.5) | 73.6 (7.0) | 0.08 | 0.06 | -0.1 | 0.21 | 0.1 | 0.17 | 0.1 |
| Ornithine | 68.6 (13.5) | 66.7 (17.9) | 0.33 | 0.48 | -0.1 | 0.34 | 0.1 | 0.27 | 0.2 |
| Aspartate | 6.3 (1.3) | 7.1 (2.1) | 0.12 | 0.08 | 0.2 | 0.84 | 0.0 | 0.94 | 0.0 |
| Glutamate | 73.0 (29.3) | 72.7 (31.3) | 0.95 | 0.82 | 0.0 | 0.35 | -0.2 | 0.31 | -0.3 |
| Lysine | 178.6 (19.2) | 175.2 (26.8) | 0.39 | 0.43 | 0.0 | 0.36 | 0.1 | 0.28 | 0.1 |
| Alanine | 340.0 (58.4) | 316.1 (63.2) | 0.03 | 0.08 | -0.1 | 0.15 | -0.2 | 0.33 | -0.1 |
| Phenylalanine | 62.9 (8.0) | 61.2 (7.7) | 0.28 | 0.35 | 0.0 | 0.22 | 0.1 | 0.11 | 0.1 |
| Isoleucine | 61.6 (12.0) | 67.4 (14.5) | 0.06 | 0.08 | 0.1 | 0.20 | 0.2 | 0.27 | 0.1 |
| Leucine | 118.3 (18.9) | 125.0 (23.2) | 0.23 | 0.23 | 0.1 | 0.01 | 0.3 | 0.02 | 0.2 |
| Proline | 153.9 (35.7) | 163.8 (43.2) | 0.37 | 0.40 | 0.1 | 0.40 | 0.1 | 0.31 | 0.2 |
| Valine | 242.5 (32.6) | 256.9 (42.8) | 0.15 | 0.16 | 0.1 | 2e-03 | 0.3 | 3e-03 | 0.3 |
| Asparagine | 55.2 (6.5) | 55.5 (9.9) | 0.85 | 0.92 | 0.0 | 0.24 | 0.1 | 0.20 | 0.1 |
| Glutamine | 579.9 (64.3) | 573.5 (80.6) | 0.57 | 0.62 | 0.0 | 0.12 | 0.1 | 0.13 | 0.1 |
| Threonine | 135.4 (23.6) | 135.1 (33.6) | 0.57 | 0.70 | 0.0 | 0.19 | 0.2 | 0.20 | 0.2 |
| Tyrosine | 64.7 (7.4) | 66.3 (9.3) | 0.49 | 0.50 | 0.0 | 0.27 | 0.1 | 0.30 | 0.1 |
| Tryptophan | 66.0 (10.9) | 64.8 (13.0) | 0.45 | 0.49 | 0.0 | 0.42 | 0.1 | 0.48 | 0.1 |
| α-ketoglutaric acid | 10.0 (1.7) | 10.3 (2.2) | 0.79 | 0.49 | 0.0 | 0.78 | 0.0 | 0.69 | 0.0 |
| 3-hydroxyisobutyrate | 17.0 (3.6) | 21.4 (6.1) | 1e-03 | 1e-03 | 0.3 | 1e-03 | 0.5 | 1e-03 | 0.5 |
| α-hydroxybutyrate | 36.5 (14.5) | 44.8 (20.2) | 0.06 | 0.06 | 0.3 | 0.57 | 0.1 | 0.98 | 0.0 |
| β-hydroxybutyrate | 59.7 (51.3) | 212.6 (224.3) | 6e-07 | 1e-07 | 1.6 | 1e-04 | 2.0 | 4e-04 | 1.8 |
| Methionine | 27.8 (2.9) | 28.7 (5.2) | 0.65 | 0.60 | 0.0 | 0.05 | 0.2 | 0.04 | 0.2 |
| Kynurenine | 2.5 (0.3) | 2.5 (0.4) | 0.43 | 0.71 | 0.0 | 0.14 | -0.1 | 0.46 | -0.1 |
|  |  |  |  |  |  |  |  |  |  |
| Tryptophan | 63.4 (11.4) | 62.4 (13.1) | 0.46 | 0.59 | 0.0 | 0.19 | 0.2 | 0.21 | 0.2 |
| Kynurenine | 2.7 (0.4) | 2.6 (0.4) | 0.30 | 0.48 | 0.0 | 0.13 | -0.1 | 0.40 | -0.1 |
| Kynurenic acid | 50.2 (17.4) | 54.8 (20.3) | 0.47 | 0.33 | 0.1 | 0.89 | 0.0 | 0.20 | 0.2 |
| Anthranilic acid | 16.1 (4.2) | 17.7 (5.6) | 0.40 | 0.18 | 0.1 | 0.36 | 0.2 | 0.21 | 0.2 |
| 3-hydroxykynurenine | 42.4 (11.8) | 48.8 (19.0) | 0.23 | 0.12 | 0.2 | 0.54 | -0.1 | 0.85 | 0.0 |
| Xanthurenic acid | 14.7 (5.4) | 16.0 (5.8) | 0.41 | 0.29 | 0.1 | 0.30 | 0.2 | 0.10 | 0.3 |
| 3-hydroxyanthranilic acid | 33.1 (11.3) | 30.4 (10.8) | 0.37 | 0.20 | -0.1 | 0.75 | 0.1 | 0.55 | 0.1 |
| Picolinic acid | 35.4 (12.1) | 53.2 (20.7) | 3e-05 | 7e-06 | 0.6 | 3e-04 | 0.8 | 1e-04 | 0.8 |
| Quinolinc acid | 375.3 (90.2) | 375.5 (156.2) | 0.20 | 0.65 | 0.0 | 0.01 | -0.5 | 0.05 | -0.3 |
| Neopterin | 10.6 (3.3) | 11.3 (3.9) | 0.43 | 0.33 | 0.1 | 0.36 | -0.2 | 0.95 | 0.0 |
| Nicotinamide | 261.0 (92.1) | 211.2 (61.3) | 0.01 | 0.01 | -0.3 | 0.51 | 0.1 | 0.51 | 0.1 |
| N1-methylnicotinamide | 151.8 (76.3) | 119.6 (71.2) | 0.02 | 0.03 | -0.4 | 0.91 | 0.0 | 0.76 | 0.1 |
| Thiamine | 4.3 (1.5) | 4.8 (3.0) | 0.89 | 0.70 | 0.1 | 0.31 | -0.3 | 0.32 | -0.3 |
| Thiamine monophosphate | 8.9 (2.4) | 9.0 (3.4) | 0.93 | 0.84 | 0.0 | 0.50 | -0.1 | 0.68 | -0.1 |
| Total thiamine* | 12.1 (3.1) | 13.2 (6.8) | 0.95 | 0.88 | 0.0 | 0.45 | -0.2 | 0.56 | -0.1 |
| Riboflavin | 13.8 (8.2) | 14.0 (8.1) | 0.78 | 0.95 | 0.0 | 0.60 | -0.2 | 0.52 | -0.2 |
| Flavin mononucleotide | 13.3 (4.0) | 12.9 (4.3) | 0.66 | 0.65 | 0.0 | 0.78 | 0.1 | 0.82 | 0.0 |
| Pyridoxal 5’-phosphate | 72.6 (46.2) | 66.8 (57.8) | 0.11 | 0.27 | -0.2 | 0.97 | 0.0 | 0.91 | 0.0 |
| Pyridoxal | 14.1 (7.8) | 13.9 (8.9) | 0.50 | 0.69 | -0.1 | 0.91 | 0.0 | 0.86 | -0.1 |
| 4-pyridoxic acid | 29.9 (14.7) | 32.0 (19.5) | 0.78 | 0.88 | 0.0 | 0.55 | -0.2 | 0.79 | -0.1 |
| Trigonelline | 4.8 (3.9) | 3.8 (2.8) | 0.34 | 0.31 | -0.2 | 0.09 | -0.7 | 0.27 | -0.4 |
| Cystathionine | 1.3 (0.1) | 1.3 (0.2) | 0.98 | 0.68 | 0.0 | 0.62 | 0.0 | 0.27 | 0.1 |


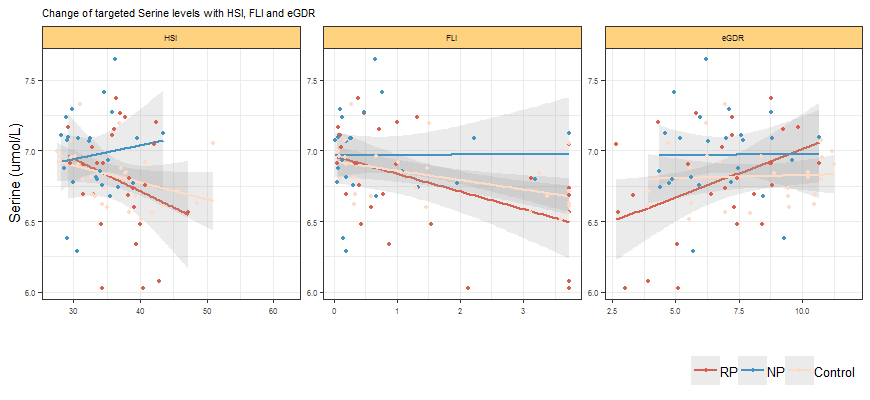


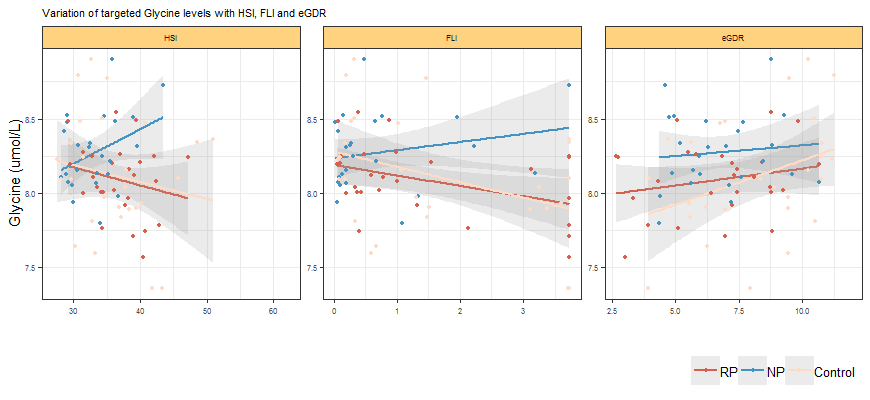


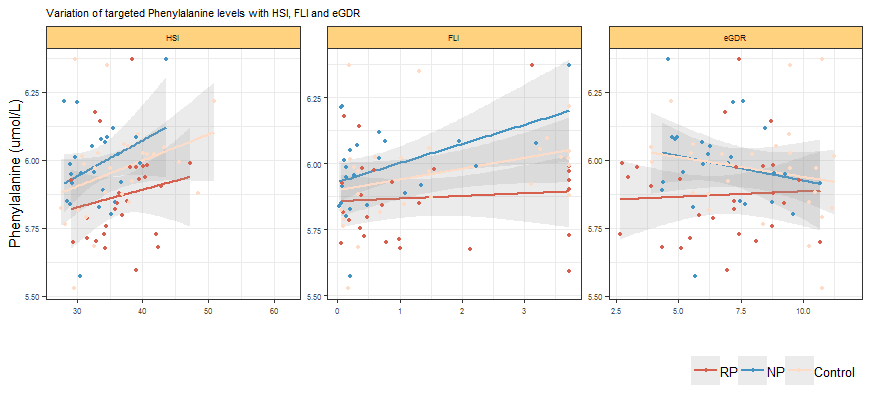


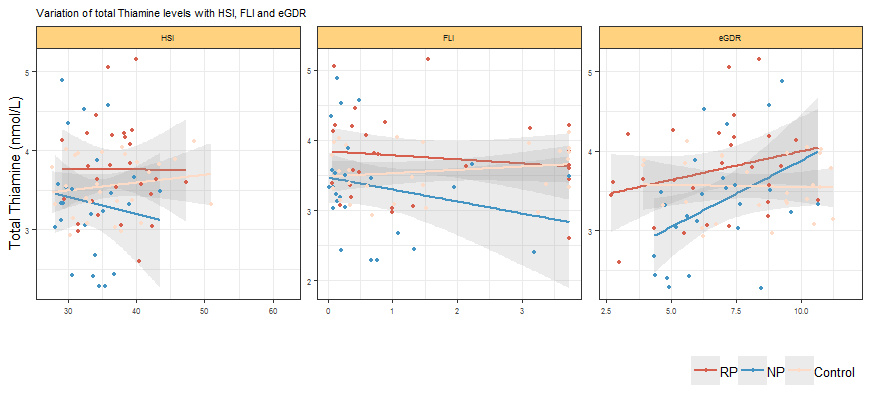


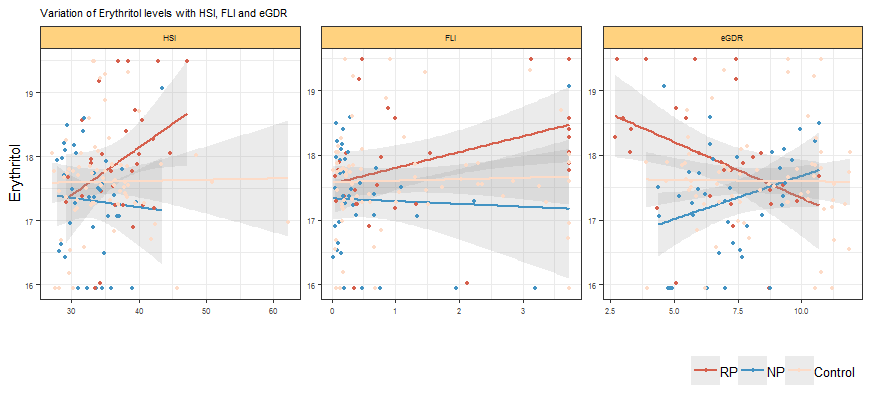


**Figure S1.** Correlation patterns of targeted serine, glycine, phenylalanine, total thiamine and erythritol levels with HSI, FLI and eGDR. Winsorized data. (Total thiamine is calculated as total of thiamine monophosphate and thiamine)
